# Identifying parameter regions for multistationarity

**DOI:** 10.1371/journal.pcbi.1005751

**Published:** 2017-10-03

**Authors:** Carsten Conradi, Elisenda Feliu, Maya Mincheva, Carsten Wiuf

**Affiliations:** 1 Life Science Engineering, HTW Berlin, Berlin, Germany; 2 Department of Mathematical Sciences, University of Copenhagen, Copenhagen, Denmark; 3 Department of Mathematical Sciences, Northern Illinois University, DeKalb, Illinois, United States of America; Oxford, UNITED KINGDOM

## Abstract

Mathematical modelling has become an established tool for studying the dynamics of biological systems. Current applications range from building models that reproduce quantitative data to identifying systems with predefined qualitative features, such as switching behaviour, bistability or oscillations. Mathematically, the latter question amounts to identifying parameter values associated with a given qualitative feature. We introduce a procedure to partition the parameter space of a parameterized system of ordinary differential equations into regions for which the system has a unique or multiple equilibria. The procedure is based on the computation of the Brouwer degree, and it creates a multivariate polynomial with parameter depending coefficients. The signs of the coefficients determine parameter regions with and without multistationarity. A particular strength of the procedure is the avoidance of numerical analysis and parameter sampling. The procedure consists of a number of steps. Each of these steps might be addressed algorithmically using various computer programs and available software, or manually. We demonstrate our procedure on several models of gene transcription and cell signalling, and show that in many cases we obtain a complete partitioning of the parameter space with respect to multistationarity.

## Author summary

Mathematical modelling has become an important tool in biology. As modelling requires separating the essential from the ordinary, there is never just one model but a collection thereof. To understand biology through modelling it is therefore crucial to be able to tell which of these models are capable of reproducing an observed behaviour and which are not. For example, to understand cellular decision making, models allowing multiple equilibria are studied and one asks which models allow for this behaviour. Here we describe a procedure that links the existence of a unique and of multiple equilibria to the sign of a single expression. We demonstrate the usefulness of the procedure by applying it to models of gene transcription and cellular signalling.

## Introduction

Mathematical models in the form of parameterized systems of ordinary differential equations (ODEs) are valuable tools in biology. Often, qualitative properties of the ODEs are associated with macroscopic biological properties and biological functions [1–4]. It is therefore important that we are able to analyse mathematical models with respect to their qualitative features and to understand when these properties arise in models. With the growing adaptation of differential equations in biology, an automated screening of ODE models for parameter dependent properties and discrimination of parameter regions with different properties would be a very useful tool for biology, and perhaps even more for synthetic biology [5]. Even though it is currently not conceivable how and if this task can be efficiently formalized, we view the procedure presented here as a first step in this direction.

*Multistationarity*, that is, the capacity of the system to rest in different positive equilibria depending on the initial state of the system, is an important qualitative property. Biologically, multistationarity is linked to cellular decision making and ‘memory’-related on/off responses to graded input [2–4]. It has been suggested that different stable equilibria of a cell represent different cell types [6, 7]. Whole-cell modelling provides an opportunity to understand the number and type of the stable equilibria of the cell and could potentially give insight into the different cell types that a particular cell can differentiate into and transition between. Currently, this is an important open question in biology [8]. Moreover, the existence of multiple equilibria is often a design objective in synthetic biology [9, 10]. Various mathematical methods, developed in the context of reaction network theory, can be applied to decide whether multistationarity exists for some parameter values or not at all, or to pinpoint specific values for which it does occur [11–20]. Some of these methods are freely available as software tools [21, 22].

It is a hard mathematical problem to delimit parameter regions for which multistationarity occurs. Often it is solved by numerical investigations and parameter sampling, guided by biological intuition or by case-by-case mathematical approaches. A general approach, in part numerical, is based on a certain bifurcation condition [18, 19, 23, 24]. Alternatively, for polynomial ODEs, a decomposition of the parameter space into regions with different numbers of equilibria could be achieved by Cylindrical Algebraic Decomposition (a version of quantifier elimination) [25]. This method, however, scales very poorly and is thus only of limited help in biology, where models tend to be large in terms of the number of variables and parameters.

Here we present two new theoretical results pertaining to multistationarity (Theorem 1 and Corollary 2). The results are in the context of reaction network theory and generalize ideas in [26, 27]. We consider a parameterized ODE system defined by a reaction network and compute a single polynomial in the species concentrations with coefficients depending on the parameters of the system. The theoretical results relate the capacity for multiple equilibria or a single equilibrium to the signs of the polynomial as a function of the parameters and the variables (concentrations).

The theoretical results apply to *dissipative* reaction networks (networks for which all trajectories eventually remain in a compact set) without *boundary equilibria* in stoichiometric compatibility classes with non-empty interior. These conditions are met in many reaction network models of molecular systems. We show by example that the results allow us to identify regions of the parameter space for which multiple equilibria exist and regions for which only one equilibrium exists. Subsequently this leads to the formulation of a general *procedure* for detecting regions of mono- and multistationarity. The procedure verifies the conditions of the theoretical results and further, calculates the before-mentioned polynomial. A key ingredient is the existence of a *positive parameterization* of the set of positive equilibria. Such a parameterization is known to exist for many classes of reaction networks, for example, systems with toric steady states [14] and post-translational modification systems [28, 29].

The conditions of the procedure might be verified manually or algorithmically according to computational criteria. The algorithmic criteria are, however, only sufficient for the conditions to hold. For example, a basic condition is that of dissipativity. To our knowledge there is not a sufficient and necessary computational criterion for dissipativity, but several sufficient ones. If these fail, then the reaction network might still be dissipative, which might be verified by other means. By collecting the algorithmic criteria, the procedure can be formulated as a fully automated procedure (an algorithm) that partitions the parameter space without any manual intervention. The algorithm might however terminate indecisively if some of the criteria are not met.

Table 1 shows two examples of reaction network motifs that occur frequently in intracellular signalling: a two-site protein modification by a kinase–phosphatase pair and a one-site modification of two proteins by the same kinase–phosphatase pair. These reaction networks are in the domain of the automated procedure and conditions for mono- and multistationarity can be found without any manual intervention. The conditions discriminating between a unique and multiple equilibria highlight a delicate relationship between the catalytic and Michaelis-Menten constants of the kinase and the phosphatase with the modified protein as a substrate (the *k*_*c*_- and *k*_*M*_-values). If the condition for multiple equilibria is met, then multiple equilibria occur provided the total concentrations of kinase, phosphatase and substrate are in suitable ranges (values thereof can be computed as part of the procedure).

**Table 1:** Conditions for unique and multiple equilibria in post-translational modification of proteins.

Motif	Condition
$A+K⇌AK⟶A_{p}+K\;B+K⇌BK⟶B_{p}+K$ $A_{p}+F⇌A_{p}F⟶A+F\;B_{p}+F⇌B_{p}F⟶B+F$	$b\left(\kappa \right)=\left(k_{c1}k_{c4}-k_{c2}k_{c3}\right)\cdot \left(\frac{k_{c1}k_{c4}}{k_{M1}k_{M4}}-\frac{k_{c2}k_{c3}}{k_{M2}k_{M3}}\right)$ Multiple: *b*(*κ*) < 0, Unique: *b*(*κ*) ≥ 0
$A+K⇌AK⟶A_{p}+K⇌A_{p}K⟶A_{pp}+K$ $A_{pp}+F⇌A_{pp}F⟶A_{p}+F⇌A_{p}F⟶A+F$	*b*_1_(*κ*) = *k*_*c*1_*k*_*c*4_ − *k*_*c*2_*k*_*c*3_ *b*_2_(*κ*) = *k*_*c*1_*k*_*c*4_(*k*_*M*2_ + *k*_*M*3_) − *k*_*c*2_*k*_*c*3_(*k*_*M*1_ + *k*_*M*4_) Multiple: *b*_1_(*κ*) < 0, Unique: *b*_1_(*κ*) ≥ 0 and *b*_2_(*κ*) ≥ 0

The symbols *k*_*ci*_ and *k*_*Mi*_ denote respectively the catalytic and the Michaelis-Menten constants of the *i*-th modification step (*i* = 1: phosphorylation of *A*, *i* = 2: dephosphorylation of *A*_*p*_, *i* = 3: phosphorylation of *B* or *A*_*p*_, *i* = 4: dephosphorylation of *B*_*p*_ or *A*_*pp*_). All parameter values satisfying the conditions in the second column yield multiple (unique) equilibria for some (all) values of the conserved quantities. For the second motif, we cannot decide on the number of equilibria for *b*_1_(*κ*) ≥ 0 and *b*_2_(*κ*) < 0. See §6.1 and §6.2 in the S1 File for details.

The paper has three main sections: a theoretical section, a section about the procedure and an application section. We close the paper with two brief sections discussing computational limitations, related work and future directions. In the theoretical section we first introduce notation and mathematical background material. We then give the theorem and the corollary that links the number of equilibria to the sign of the determinant of the Jacobian of a certain function, which is derived from the ODE system associated with a reaction network. In the second section we state the procedure, derive the algorithm and comment on the feasibility and verifiability of the conditions. Finally, in the application section we apply the procedure to several examples. The S1 File has six sections. All proofs are relegated to §1–4 together with background material. In §5 we elaborate further on how the conditions of the procedure/algorithm can be verified. In §6 we provide details of the algorithmic analysis of the examples in Table 1. Also we include a further monostationary example for illustration of the algorithm.

## Results

### Theory

In this part of the manuscript we present the theoretical results. We start by introducing the basic formalism of reaction networks. Theorem 1, Corollary 1 and 2 below apply to *dissipative networks* without *boundary equilibria* and concern the (non)existence of *multiple equilibria* in some *stoichiometric compatibility class*. Corollary 2 assumes the existence of a *positive parameterization* of the set of positive equilibria. Before stating the results these five concepts are formally defined.

#### Reaction networks

**Fig 1:**
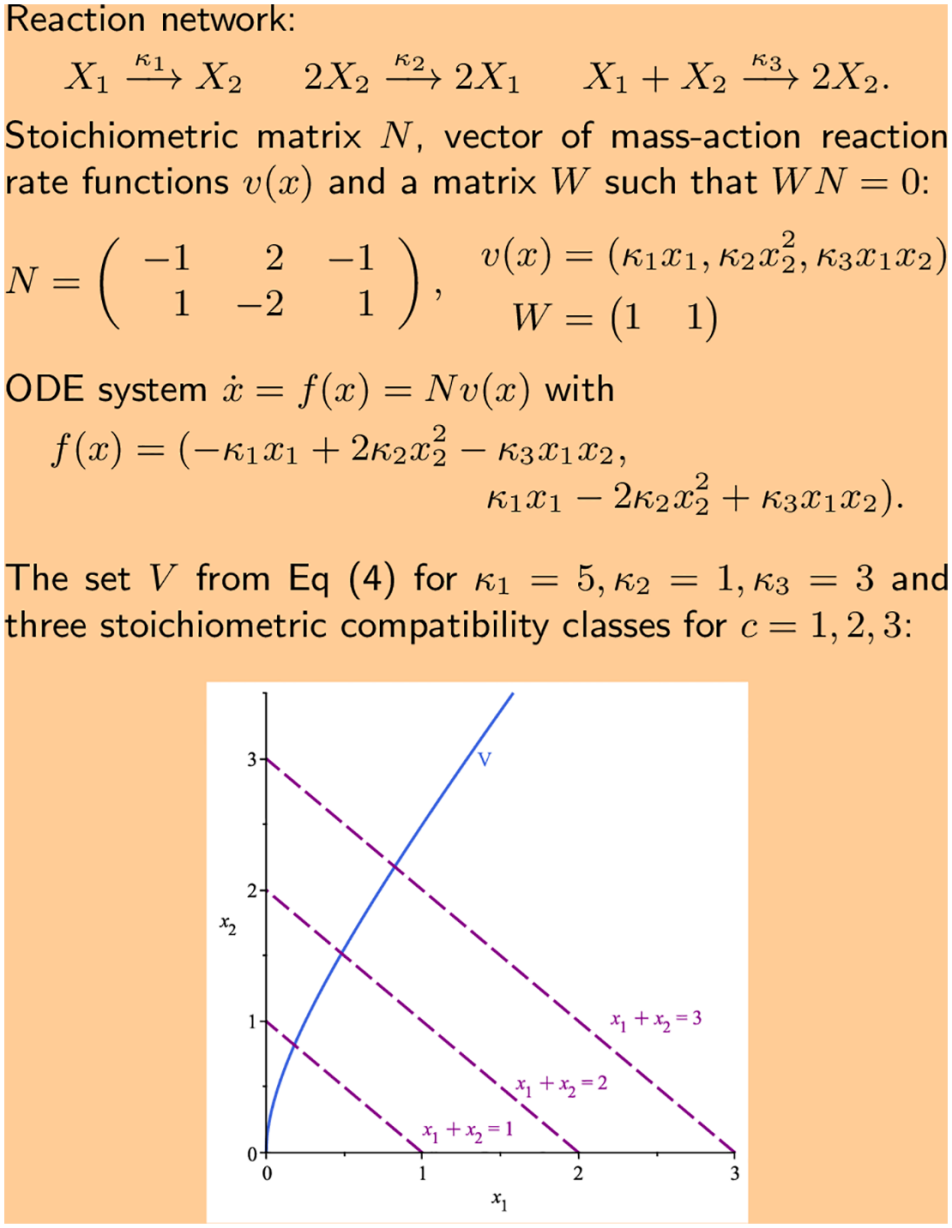
Running example. Example network with two species, *X*_1_ and *X*_2_, and three reactions with mass-action kinetics.

A *reaction network*, or simply a *network*, consists of a set of species {*X*_1_, …, *X*_*n*_} and a set of reactions of the form:

$$\begin{array}{c} R_{j}:\sum_{i=1}^{n}\alpha_{ij}X_{i}⟶\sum_{i=1}^{n}\beta_{ij}X_{i},\;j=1,\cdots ,\ell \end{array}$$ (1)

where *α*_*ij*_, *β*_*ij*_ are non-negative integers. The left hand side is called the reactant, while the right hand side is called the product. We let $N=\left(N_{ij}\right)\in R^{n\times \ell }$ be the *stoichiometric matrix* of the network, defined as *N*_*ij*_ = *β*_*ij*_ − *α*_*ij*_, that is, the (*i*, *j*)-th entry encodes the net production of species *X*_*i*_ in reaction *R*_*j*_. We refer to the ‘running example’ in Fig 1 for an illustration of the definitions.

The concentrations of the species *X*_1_, …, *X*_*n*_ are denoted by lower-case letters *x*_1_, …, *x*_*n*_ and we let *x* = (*x*_1_, …, *x*_*n*_). We denote by $R_{>0}^{n}$ ($R_{\geq 0}^{n}$), the positive (non-negative) orthant in $R^{n}$. The evolution of the concentrations with respect to time is modeled as an ODE system derived from a set of *reaction rate functions*. A reaction rate function for reaction *R*_*j*_ is a $C^{1}$-function $v_{j}:R_{\geq 0}^{n}⟶R_{\geq 0}$ that models the (non-negative) speed of the reaction. We further assume that

$$\begin{array}{c} v_{j}\left(x\right)=0\;\Leftrightarrow \;x_{i}=0\;\text{for}\;\text{some}\;i\;\text{such}\;\text{that}\;\alpha_{ij}>0, \end{array}$$ (2)

that is, the reaction only takes place in the presence of all reactant species. We refer to the set of reaction rate functions as the *kinetics*.

A particular important example of a kinetics is that of *mass-action kinetics*. In this case the reaction rate functions are given by

$$\begin{array}{c} v_{j}\left(x\right)=\kappa_{j}x_{1}^{\alpha_{1j}}\cdot \ldots \cdot x_{n}^{\alpha_{nj}},\;j=1,\cdots ,\ell , \end{array}$$

where *κ*_*j*_ is a positive number called the *reaction rate constant* and we assume 0^0^ = 1. Other important examples are Michaelis-Menten kinetics and Hill kinetics. All three types of kinetics fulfil the assumption in Eq (2).

For a choice of reaction rate functions *v* = (*v*_1_, …, *v*_*ℓ*_), the ODE system modelling the species concentrations over time with initial condition *x*(0) = *x*_0_, is

$$\begin{array}{c} \dot{x}=f\left(x\right),\;x\in R_{\geq 0}^{n},\;\text{where}\;f\left(x\right)=Nv\left(x\right). \end{array}$$ (3)

Under assumption Eq (2), the orthants $R_{>0}^{n}$ and $R_{\geq 0}^{n}$ are *forward-invariant* under *f* in Eq (3) [30, Theorem 5.6], [31, Section 16]. Forward-invariance implies that the solutions to the ODE system stays in $R_{>0}^{n}$ (resp. $R_{\geq 0}^{n}$) for all positive times if the initial condition is in $R_{>0}^{n}$ (resp. $R_{\geq 0}^{n}$).

The trajectories of the ODEs in Eq (3) are confined to the so-called *stoichiometric compatibility classes*, which are defined as follows. Let *s* = rank(*N*) be the rank of the network and *d* = *n* − *s* be the corank. Further, let $W\in R^{d\times n}$ be any matrix of full rank *d* such that *WN* = 0, see Fig 1 for an example. This matrix is zero-dimensional if *N* has full rank *n*. For each $c\in R^{d}$, there is an associated *stoichiometric compatibility class* defined as

$$\begin{array}{c} P_{c}:=\left\{x\in R_{\geq 0}^{n}|Wx=c\right\}. \end{array}$$

This set is empty if $c\notin W(R_{\geq 0}^{n})$. The *positive stoichiometric compatibility class* is defined as the relative interior of $P_{c}$, that is, the intersection of $P_{c}$ with the positive orthant:

$$\begin{array}{c} P_{c}^{+}:=\left\{x\in R_{>0}^{n}|Wx=c\right\}=P_{c}\cap R_{>0}^{n}. \end{array}$$

The sets $P_{c}^{+}$ and $P_{c}$ are convex. Since by construction *Wx* is conserved over time and determined by the initial condition, then $P_{c}^{+}$ and $P_{c}$ are also forward-invariant.

An equation of the form *ω* ⋅ *x* = *c*′ for some *ω* ∈ im(*N*)^⊥^ and $c^{\prime }\in R$ is called a *conservation relation*. In particular, *Wx* = *c* forms a system of *d* conservation relations.

For the running example in Fig 1, the rank of the network is *s* = 1 and the corank is *d* = 1. The matrix *W* in the figure leads to the conservation relation *x*_1_ + *x*_2_ = *c*. Here the stoichiometric compatibility class $P_{c}$ has non-empty interior, that is, $P_{c}^{+}\neq \emptyset$, if and only if *c* > 0.

In the following, to ease the notation, we implicitly assume a reaction network comes with a kinetics (a set of reaction rate functions) and the associated ODE system.

#### Dissipative and conservative reaction networks

A reaction network is *dissipative* if, for all stoichiometric compatibility classes $P_{c}$, there exists a compact set where the trajectories of $P_{c}$ eventually enter (see §3.2 in the S1 File). A reaction network is *conservative* if there exists a conservation relation with only positive coefficients, or, equivalently, if for all species *X*_*i*_ there is a conservation relation such that the coefficient of *x*_*i*_ is positive and all other coefficients are non-negative. This is equivalent to the stoichiometric compatibility classes being compact sets [32]. Hence, in particular, a conservative reaction network is dissipative because we can choose the attracting compact set to be the stoichiometric compatibility class itself. Because of the conservation relation *x*_1_ + *x*_2_ = *c*, the reaction network of the running example is conservative.

#### Equilibria

Given the ODE in Eq (3), the set of non-negative equilibria is the set of points for which *f*(*x*) vanishes:

$$\begin{array}{c} V=\{x\in R_{\geq 0}^{n}|f\left(x\right)=0\}. \end{array}$$ (4)

We are interested in the positive equilibria in each stoichiometric compatibility class, that is, in the set $V\cap P_{c}^{+}$. Generically, this set consists of isolated points obtained as the simultaneous positive solutions to the equations

$$\begin{array}{c} f(x)=0,\;Wx=c. \end{array}$$ (5)

Fig 1 shows a representation of the set *V* together with examples of stoichiometric compatibility classes for the running example. The figure suggests that the set *V* intersects each stoichiometric compatibility class in exactly one point.

We introduce some definitions: a network admits *multiple equilibria* (or is *multistationary*) if there exists $c\in R^{d}$ such that $V\cap P_{c}^{+}$ contains at least two points, that is, the system in Eq (5) has at least two positive solutions. Equilibria belonging to $V\cap P_{c}$ but not to $V\cap P_{c}^{+}$ for some *c* are *boundary equilibria*. A boundary equilibrium has at least one coordinate equal to zero.

#### The function *φ*_*c*_(*x*)

Some of the *n* equations in the system *f*(*x*) = 0 might be redundant. Indeed, every vector *ω* ∈ im(*N*)^⊥^ fulfils *ω* ⋅ *f*(*x*) = 0, and hence gives a linear relation among the entries of *f*(*x*). As a consequence, there are (at least) as many independent linear relations as rows of *W*, that is, *d*, and there are at most *s* = *n* − *d* linearly independent equations in the system *f*(*x*) = 0. Thus *d* of the equations are redundant. By removing these from *f*(*x*) = 0, the system in Eq (5) becomes a system of *n* equations in *n* variables.

In order to systematically choose *d* equations to remove, we proceed as follows. We choose the matrix of conservation relations $W\in R^{d\times n}$ to be row reduced and let *i*_1_, …, *i*_*d*_ be the indices of the first non-zero coordinate of each row. Then the scalar product of the *j*-th row of *W* with *f*(*x*) can be used to express *f*_*i*_*j*__(*x*) as a linear combination of the entries of *f*(*x*) with indices different from *i*_1_, …, *i*_*d*_. It follows that the equations *f*_*i*_1__(*x*) = 0, …, *f*_*i*_*d*__(*x*) = 0 can be removed.

For $c\in R^{d}$, we define the $C^{1}$-function $\varphi_{c}\left(x\right):R_{\geq 0}^{n}⟶R^{n}$ by

$$\begin{array}{c} \varphi_{c}{\left(x\right)}_{i}=\left\{ \begin{array}{cc} f_{i}\left(x\right) & i\notin \{i_{1},\cdots ,i_{d}\}\\ {\left(Wx-c\right)}_{i} & i\in \{i_{1},\cdots ,i_{d}\}. \end{array}\right. \end{array}$$ (6)

For the running example in Fig 1 the matrix *W* is already row reduced with *i*_1_ = 1. Hence *φ*_*c*_ is obtained by replacing *f*_1_(*x*) with *x*_1_ + *x*_2_ − *c*:

$$\begin{array}{c} \varphi_{c}\left(x\right)=(\begin{array}{c} x_{1}+x_{2}-c\\ \kappa_{1}x_{1}-2\kappa_{2}x_{2}^{2}+\kappa_{3}x_{1}x_{2} \end{array}). \end{array}$$

As the function *φ*_*c*_(*x*) is obtained by replacing redundant equations in *f*(*x*) = 0 with equations defining $P_{c}$, we have

$$\begin{array}{c} V\cap P_{c}=\left\{x\in R_{\geq 0}^{n}|\varphi_{c}\left(x\right)=0\right\}. \end{array}$$

Consequently, a network admits *multiple equilibria* if the equation *φ*_*c*_(*x*) = 0 has at least two positive solutions for some $c\in R^{d}$.

#### A theorem for unique and multiple equilibria

Let $M\left(x\right)\in R^{n\times n}$ be the Jacobian matrix of *φ*_*c*_(*x*), that is, the matrix with (*i*, *j*)-th entry equal to the partial derivative of *φ*_*c*,*i*_(*x*) with respect to *x*_*j*_. The matrix *M*(*x*) does not depend on *c*, see Eq (6).

We say that an equilibrium $x^{*}\in V\cap P_{c}$ is *non-degenerate* if the Jacobian of *φ*_*c*_ at x*, *M*(x*), is non-singular, that is, if det(*M*(x*)) ≠ 0 [13].

**Theorem 1 (Unique and multiple equilibria).** Assume the reaction rate functions fulfil Eq (2), let *s* = rank(*N*) and let $P_{c}$ be a stoichiometric compatibility class such that $P_{c}^{+}\neq \emptyset$, where $c\in R^{d}$. Further, assume that

(i) The network is dissipative.

(ii) There are no boundary equilibria in $P_{c}$.

Then the following holds.

(A’) **Uniqueness of equilibria.** If

$$\begin{array}{c} \text{sign}\left(\text{det}\left(M\left(x\right)\right)\right)={\left(-1\right)}^{s}\;\text{for}\;\text{all}\;\text{positive}\;\text{equilibria}\;x\in V\cap P_{c}^{+}, \end{array}$$

then there is exactly one positive equilibrium in $P_{c}$. Further, this equilibrium is non-degenerate.

(B’) **Multiple equilibria.** If

$$\begin{array}{c} \text{sign}\left(\text{det}\left(M\left(x\right)\right)\right)={\left(-1\right)}^{s+1}\;\;\text{for}\;\text{some}\;\text{equilibrium}\;\;x\in V\cap P_{c}^{+}, \end{array}$$

then there are at least two positive equilibria in $P_{c}$, at least one of which is non-degenerate. If all positive equilibria in $P_{c}$ are non-degenerate, then there are at least three and always an odd number.

The proof of Theorem 1 is based on relating det(*M*(*x*)) to the Brouwer degree of *φ*_*c*_ at 0 (see §1-§4 in the S1 File). Note that the only situation that is not covered by Theorem 1 is when sign(det(*M*(*x*))) takes the value 0 for some *x*, but never the value (−1)^*s*+1^. The determinant of *M*(*x*) is the same as the *core determinant* in [33, Lemma 3.7]. See also [13, Remark 9.27].

To check whether the sign conditions in part (A’) or (B’) hold requires information about the equilibria in $P_{c}^{+}$. As such, these conditions are difficult to check. If sign(det(*M*(*x*))) is constant for all *x* in a set containing the positive equilibria, then the condition in (A’) is always fulfilled. In particular, this is the case for *injective networks*, where sign(det(*M*(*x*))) = (−1)^*s*^ for all $x\in R_{>0}^{n}$ [13] (see also [34–39] for related work on injective networks). The latter might be verified or falsified without any knowledge about the equilibria of the system (see the comments to Step 5 and Step 7 in the section “Procedure for finding parameter regions for mono- and multistationarity”).

**Corollary 1 (Unique equilibria).** Assume that the assumptions of Theorem 1 hold and that sign(det(*M*(*x*))) = (−1)^*s*^ for all $x\in R_{>0}^{n}$. Then there is exactly one positive equilibrium in each stoichiometric compatibility class. Further, this equilibrium is non-degenerate.

The conclusions of Theorem 1 refer specifically to non-degenerate equilibria. Non-degenerate equilibria are always isolated from each other within a given stoichiometric compatibility class, as det(*M*(*x*)) ≠ 0 ensures *M*(*x*) is locally invertible. In some situations we might be able to “lift” non-degenerate equilibria of a reaction network to another reaction network that in some sense is larger, thereby proving lower bounds on the number of non-degenerate equilibria of the larger reaction network. This is for example the case if the smaller network is embedded in the larger [40, 41], if the smaller network is without inflows/outflows while the larger has all inflows/outflows [42], or if the smaller is obtained by elimination of intermediate species [43]. Conditions for the existence of degenerate equilibria, where det(*M*(*x*)) is expected to change sign, are also known [23, 44].

#### Positive parameterizations and a corollary

Verifying condition (A’) or (B’) is considerably easier if there exists a positive parameterization of the set $V\cap R_{>0}^{n}$ of all positive equilibria. In this subsection we define such a parameterization and restate Theorem 1 as Corollary 2 in this situation. In the following sections this corollary will become the foundation for the procedure to partition the parameter space into regions with different equilibrium properties.

By a *positive parameterization* of the set of positive equilibria we mean a surjective function

$$\begin{array}{c} \begin{array}{cccc} \Phi : & R_{>0}^{m} & ⟶ & V\cap R_{>0}^{n}\\ & \hat{x}=\left({\hat{x}}_{1},\cdots ,{\hat{x}}_{m}\right) & \mapsto & (\Phi_{1}\left(\hat{x}\right),\cdots ,\Phi_{n}\left(\hat{x}\right)), \end{array} \end{array}$$ (7)

for some *m* < *n*, such that $\hat{x}\in R_{>0}^{m}$ is the vector of free variables. In other words, a positive parameterization implies that *x*_1_, …, *x*_*n*_ are expressed at equilibrium as functions of $\hat{x}$:

$$\begin{array}{c} x_{i}=\Phi_{i}\left(\hat{x}\right),\;i=1,\cdots ,n, \end{array}$$

such that *x*_1_, …, *x*_*n*_ are positive provided $\hat{x}$ is positive. Thus

$$\begin{array}{c} V\cap R_{>0}^{n}=\left\{\Phi \left(\hat{x}\right)|\hat{x}\in R_{>0}^{m}\right\}. \end{array}$$ (8)

Typically, the number of free variables equals the corank of the network, that is *m* = *d* = *n* − *s*.

**Fig 2:**
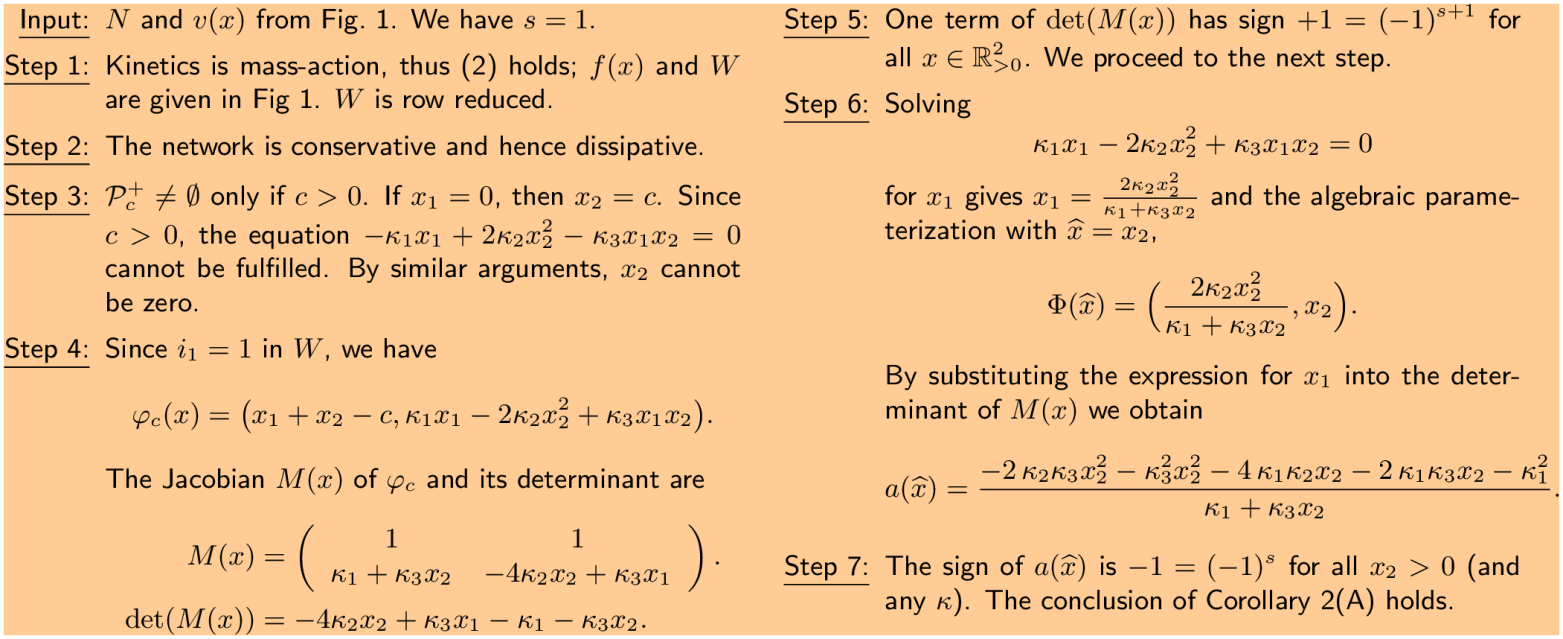
Step 1-3 check the assumptions of Corollary 1 and Corollary 2. In step 4 the function *φ*_*c*_(*x*) is constructed and the determinant of *M*(*x*) is found. Step 5 is the sign analysis of the polynomial det(*M*(*x*)) for $x\in R_{>0}^{2}$. Step 6 establishes a positive parameterization and finds the polynomial $a(\hat{x})$. Step 7 is similar to step 5, but for $a(\hat{x})$.

We say that a parameterization is *algebraic* if the components $\Phi_{i}\left(\hat{x}\right)$ are polynomials or rational functions (quotients of polynomials) and can be given such that the denominator is positive for all $\hat{x}$. See Fig 2 (Step 6) for an application to the running example. Note that the parameterizations considered here do not make use of the conservation relations.

A positive equilibrium $\Phi (\hat{x})$, $\hat{x}\in R_{>0}^{m}$, belongs to the stoichiometric compatibility class $P_{c}$ where

$$\begin{array}{c} c:=W\Phi (\hat{x}). \end{array}$$ (9)

Combining Eqs (8) and (9), it follows that the positive solutions to Eq (5) for a given *c* are in one-to-one correspondence with the positive solutions to Eq (9), that is,

$$\begin{array}{c} V\cap P_{c}^{+}=\left\{\Phi \left(\hat{x}\right)|\hat{x}\in R_{>0}^{m}\;\text{and}\;c=W\Phi \left(\hat{x}\right)\right\}. \end{array}$$

In order to restate Theorem 1 using the parameterization Φ, we consider the determinant of *M*(*x*) evaluated at $\Phi (\hat{x})$,

$$\begin{array}{c} a\left(\hat{x}\right)=\text{det}\left(M\left(\Phi \left(\hat{x}\right)\right)\right),\;\hat{x}\in R_{>0}^{m}. \end{array}$$ (10)

**Corollary 2 (Positive parameterization).** Assume the reaction rate functions fulfil Eq (2) and let *s* = rank(*N*). Further, assume that

(i) The network is dissipative.

(ii) There are no boundary equilibria in $P_{c}$, for all $c\in R^{d}$ such that $P_{c}^{+}\neq \emptyset$.

(iii) The set of positive equilibria admits a positive parameterization as in Eq (7).

Then the following holds.

(A) **Uniqueness of equilibria.** If

$$\begin{array}{c} \text{sign}\left(a\left(\hat{x}\right)\right)={\left(-1\right)}^{s}\;\text{for}\;\text{all}\;\hat{x}\in R_{>0}^{m}, \end{array}$$

then there is exactly one positive equilibrium in each $P_{c}$ with $P_{c}^{+}\neq \emptyset$. Further, this equilibrium is non-degenerate.

(B) **Multiple equilibria.** If

$$\begin{array}{c} \text{sign}\left(a\left(\hat{x}\right)\right)={\left(-1\right)}^{s+1}\;\text{for}\;\text{some}\;\hat{x}\in R_{>0}^{m}, \end{array}$$

then there are at least two positive equilibria in the stoichiometric compatibility class $P_{c}$ where $c:=W\Phi (\hat{x})$. Further, at least one of the equilibria is non-degenerate. If all positive equilibria in $P_{c}$ are non-degenerate, then there are at least three equilibria and always an odd number.

Note that, contrary to Theorem 1, the stoichiometric compatibility class $P_{c}$ is not fixed in the corollary.

In the next section we formulate a procedure based on Corollary 1 and Corollary 2 to find regions of mono- and multistationarity. Before that we end this section with an application to the running example. The analysis is divided into seven steps which prelude the steps of the procedure.

#### Application of Corollary 1 and Corollary 2 to the running example

We start with the setup given in Fig 1 and first check whether the sign condition of Corollary 1 is fulfilled, in which case there is a single equilibrium in all stoichiometric compatibility classes. The steps of the analysis are illustrated in Fig 2.

The assumptions of the corollary are easily verified in this case. As we are assuming mass-action kinetics, Eq (2) is fulfilled (Fig 2, Step 1). Further the network is conservative, hence dissipative and (i) is fulfilled (Fig 2, Step 2). It is easily seen that there are no boundary equilibria in any stoichiometric compatibility class with non-empty interior (Fig 2, Step 3). Hence (ii) is fulfilled. We then construct *φ*_*c*_(*x*) and calculate the determinant of *M*(*x*). It is a polynomial (in fact, a linear function) in *x*_1_, *x*_2_ with coefficients containing both positive and negative terms (Fig 2, Step 4). By choosing $\left(x_{1},x_{2}\right)\in R_{>0}^{2}$ with *x*_1_ large enough, the determinant of *M*(*x*) is positive (Fig 2, Step 5). Therefore Corollary 1 cannot be applied as *s* = 1. We note that this conclusion is independent of the specific choice of the parameter vector *κ* = (*κ*_1_, *κ*_2_, *κ*_3_), so in fact it holds for all parameter values.

Corollary 2 has the same assumptions as Corollary 1. We find a positive parameterization by solving the equilibrium equation for *x*_1_. That is, we treat it as an equation in *x*_1_, while *x*_2_ $\left(=\hat{x}\right)$ is treated as a parameter. The function *a*(*x*_2_) obtained by substituting *x* by Φ(*x*_2_) in the determinant of *M*(*x*) is given in Fig 2, Step 6. It is clear from the expression of *a*(*x*_2_) that it takes the sign −1 for all *x*_2_ > 0. Also this conclusion does not depend on the specific value of *κ*. By application of Corollary 2(A) with *s* = 1, we conclude that there exists a unique positive non-degenerate equilibrium in each stoichiometric compatibility class with *c* > 0, for all values of the reaction rate constants (Fig 2, Step 7). The possibility of multiple equilibria is therefore excluded. In this particular example the existence of a positive parameterization is essential to draw the conclusion.

To illustrate how Corollary 2 can be used to find parameter regions for multistationarity, we consider the polynomial $a(\hat{x})$ corresponding to the hybrid histidine kinase example worked out in detail below, where *n* = 6 and *s* = 4:

$$\begin{array}{ccc} a(\hat{x}) & = & \frac{1}{\kappa_{3}}(\kappa_{2}\kappa_{4}\kappa_{5}^{2}\left(\kappa_{1}-\kappa_{3}\right)x_{4}x_{5}^{2}+\left(\kappa_{1}+\kappa_{2}\right)\kappa_{3}\kappa_{4}\kappa_{5}\kappa_{6}x_{5}^{2}+2\kappa_{1}\kappa_{2}\kappa_{3}\kappa_{4}\kappa_{5}x_{4}x_{5}\\ & & +\kappa_{1}\left(\kappa_{2}+\kappa_{3}\right)\kappa_{3}\kappa_{5}\kappa_{6}x_{5}+\kappa_{1}\kappa_{2}\kappa_{3}^{2}\kappa_{5}x_{4}+\kappa_{1}\kappa_{2}\kappa_{3}^{2}\kappa_{6}). \end{array}$$

Only one of the coefficients of the polynomial $a(\hat{x})$ in *x*_4_, *x*_5_ can be negative. If *κ*_3_ ≤ *κ*_1_, then $\text{sign}\left(a\left(\hat{x}\right)\right)={\left(-1\right)}^{4}=1$ for all positive $\hat{x}$. Corollary 2(A) implies that there is a unique positive non-degenerate equilibrium in each stoichiometric compatibility class with non-empty positive part.

Oppositely, we show that for *κ*_3_ > *κ*_1_, Corollary 2(B) applies. For that, let *x*_4_ = *T* and *x*_5_ = *T*. Then $a(\hat{x})$ becomes a polynomial in *T* with negative leading coefficient of degree 3. For *T* large enough, $a(\hat{x})$ is negative, and we conclude that there exists $\hat{x}$ such that $\text{sign}\left(a\left(\hat{x}\right)\right)={\left(-1\right)}^{5}=-1$. Corollary 2(B) implies that there exists a stoichiometric compatibility class $P_{c}$ that contains at least two positive equilibria. In summary, the region of the parameter space for which multistationarity exists is completely characterized by the inequality *κ*_3_ > *κ*_1_.

Step 5 and 7 are sign analyses of det(*M*(*x*)) and $a(\hat{x})$, respectively. These are crucial steps and essential for determining parameter regions with mono- and multistationarity. In general, the sign of a polynomial might be studied by studying the signs of the coefficients of the monomials in the polynomial. If all coefficients have the same sign, then the polynomial is either positive or negative for all $x\in R_{>0}^{n}$, respectively, $\hat{x}\in R_{>0}^{m}$, depending on the sign, and Corollary 1, respectively, Corollary 2(A) applies. If this is not the case, then Corollary 2(B) might be applicable if we can show that the polynomial has the sign (−1)^*s*+1^ for some $\hat{x}\in R_{>0}^{m}$. In the two examples discussed here, the signs of det(*M*(*x*)) and $a(\hat{x})$ are straightforward to analyse. However, this is not always the case, see the section “Checking the steps of the procedure”.

### Procedure for finding parameter regions for multistationarity

In the previous subsection we applied Corollary 1 and Corollary 2 to the running example by going through a number of steps corresponding to the conditions of the statements and the calculation of the determinant. In this section we outline the steps formally. Afterwards we discuss the steps and how they can be verified either manually or algorithmically, that is, without user intervention. Finally we devise an algorithm to conclude uniqueness of equilibria or to find regions in the parameter space where multistationarity occurs. We conclude this section with some extra examples that follow the steps of the procedure.

We assume the reaction rate functions *v*(*x*) depend on some parameters *κ*. The reaction rate functions are further assumed to be polynomials (as for mass-action kinetics) or quotients of polynomials (as for Michaelis-Menten and Hill kinetics with integer exponents).

The input to the procedure is *v*(*x*) and *N* (the stoichiometric matrix) and the output is parameter regions for which the network admits multistationarity or uniqueness of equilibria.

**Procedure (Identification of parameter regions for multistationarity)**

**Input:** *N* and *v*(*x*) depending on *κ*.

**1.** Find *f*(*x*), a row reduced matrix *W* of size *d* × *n* such that *WN* = 0, and check that *v*(*x*) vanishes in the absence of one of the reactant species, that is, check that it satisfies Eq (2).

**2.** Check that the network is dissipative.

**3.** Check for boundary equilibria in $P_{c}$ for $P_{c}^{+}\neq \emptyset$ and $c\in R^{d}$.

**4.** Construct *φ*_*c*_(*x*), *M*(*x*) and compute det(*M*(*x*)).

**5.** Analyze the sign of det(*M*(*x*)). Find conditions on the parameters *κ* such that sign(det(*M*(*x*))) = (−1)^*s*^ for all $x\in R_{>0}^{n}$, in which case Corollary 1 holds.

If Corollary 1 does not hold for all *κ*, continue to the next step.

**6.** Obtain an **algebraic** parameterization $\Phi (\hat{x})$ of the set of positive equilibria for all *κ*, as in Eq (7), such that the coefficients of the numerator and the denominator of each $\Phi_{i}\left(\hat{x}\right)$ possibly depend on *κ*. Compute $a\left(\hat{x}\right)=\text{det}\left(M\left(\Phi \left(\hat{x}\right)\right)\right)$. By hypothesis, $a(\hat{x})$ can be written as the quotient of two polynomials in $\hat{x}$ with coefficients depending on *κ*, whose denominator takes positive values.

**7.** Analyze the sign of the numerator of $a(\hat{x})$.

**7a.** Identify coefficients with sign (−1)^*s*+1^ and coefficients that can have different signs depending on the parameters.

**7b.** Use the terms corresponding to identified coefficients to construct parameter inequalities such that, whenever these inequalities hold, one has either $\text{sign}\left(a\left(\hat{x}\right)\right)={\left(-1\right)}^{s}$ for all $\hat{x}\in R_{>0}^{m}$ or $\text{sign}\left(a\left(\hat{x}\right)\right)={\left(-1\right)}^{s+1}$ for at least one $\hat{x}\in R_{>0}^{m}$, in which case either Corollary 2(A) or (B) holds.

There is no guarantee that all steps of the procedure can be carried out successfully, let alone automatically. While step 1 and 4 usually are straightforward (only computational issues might arise for large networks), step 2, 3, 5, 6 and 7 might in particular require case specific approaches. However, there exist computationally feasible sufficient criteria that guarantee the conditions in each step can be checked efficiently.

#### Checking the steps of the procedure

***Step 2: establishing dissipativity.*** If the network is not dissipative, then at least one concentration grows to infinity over time. This is typically not the case for realistic networks, but it needs to be ruled out in order to apply the procedure.

We start by checking whether the network is conservative. This implies solving the linear system *ω*^*t*^ *N* = 0 with the constraint *ω* > 0. Alternatively, conservation relations are often easily established by inspection of the reactions. For example, in many signalling networks, the total concentration of enzyme (free and bounded) and of substrate (phosphoforms) are conserved.

If the network is not conservative, then we check whether it is *strongly endotactic* [45, 46]. Strongly endotactic reaction networks are in particular *permanent*, that is, dissipative and the compact set can be chosen such that it does not intersect the boundary of $R_{>0}^{n}$, see [45–47] for details.

If the network is neither conservative nor strongly endotactic, then we can use the following proposition to decide on dissipativity (see the §3.2 in the S1 File).

**Proposition 1 (Dissipative network).** Let ||⋅|| be a norm in $R^{n}$. Assume that for each *c* with $P_{c}^{+}\neq \emptyset$, there exists a vector $\omega_{c}\in R_{>0}^{n}$ and a number *R* > 0 such that *ω*_*c*_ ⋅ *f*(*x*) < 0 for all $x\in P_{c}$ with ||*x*|| > *R*. Then the network is dissipative.

Thus, we look for vectors *ω*_*c*_ with all coordinates positive and such that *ω*_*c*_ ⋅ *f*(*x*) < 0 for large *x*. To avoid restricting the parameter values, this computation should be done symbolically.

***Step 3: absence of boundary equilibria.*** For systems of moderate size it is often possible to establish nonexistence of boundary equilibria by arguments similar to those employed in the analysis of the running example: for each *i*, assume *x*_*i*_ = 0, and show that it leads to a contradiction.

A systematic procedure to check for the existence of boundary equilibria relies on computing the so-called *minimal siphons* of the network [48]. A *siphon* is a set of species *Z* ⊆ {*X*_1_, …, *X*_*n*_} fulfilling the following closure property: if *X*_*i*_ ∈ *Z* and *X*_*i*_ is produced in reaction *R*_*j*_ (that is, *β*_*ij*_ > 0), then there exists *X*_*k*_ ∈ *Z* such that *X*_*k*_ is consumed in the same reaction (that is, *α*_*kj*_ > 0). A *minimal siphon* is a siphon that does not properly contain any other siphon.

**Proposition 2 (Siphons)** ([49, 50]) If for every minimal siphon *Z* there exists a subset {*X*_*i*_1__, …, *X*_*i*_*k*__} ⊆ *Z*, and a conservation relation λ_1_*x*_*i*_1__ + … + λ_*k*_*x*_*i*_*k*__ = *c* for some positive λ_1_, …, λ_*k*_, then the network has no boundary equilibria in any stoichiometric compatibility class $P_{c}$ with $P_{c}^{+}\neq \emptyset$.

The hypothesis of the proposition can be summarised by saying that each minimal siphon contains the support of a positive conservation relation.

For example, the running example has only one minimal siphon, namely {*X*_1_, *X*_2_}. The conservation relation *x*_1_ + *x*_2_ = *c* fulfils the requirement of Proposition 2, and hence the network has no boundary equilibria in any $P_{c}$ with *c* > 0.

More information about using siphons to preclude boundary equilibria is given in the section “Computational issues” below and in §5.1 of the S1 File.

***Step 5: determining the sign of*** det(*M*(*x*)). If the kinetics is mass-action, then det(*M*(*x*)) is a polynomial in *x*. In general, if the reaction rate functions are rational functions in *x*, then so is det(*M*(*x*)). In the latter case, if the *j*th reaction rate function fulfils *v*_*j*_(*x*) = *p*_*j*_(*x*)/*q*_*j*_(*x*) with *p*_*j*_(*x*) ≥ 0 and *q*_*j*_(*x*) > 0 for all $x\in R_{>0}^{n}$, then det(*M*(*x*)) = *p*(*x*)/*q*(*x*), where $q\left(x\right)=\prod_{j=1}^{\ell }q_{j}{\left(x\right)}^{2}>0$. It follows from the definition of *M*(*x*) and by differentiation of *v*_*j*_(*x*), *j* = 1, …, *ℓ*.

We determine conditions on the parameters such that all coefficients of *p*(*x*) have sign (−1)^*s*^. Then the sign of det(*M*(*x*)) is also (−1)^*s*^ for all $x\in R_{>0}^{n}$ and Corollary 1 holds.

***Step 6: finding an algebraic positive parameterization.*** Computer algebra systems like Maple or Mathematica can be used to find a parameterization. One strategy is to solve the equations *f*_*i*_(*x*) = 0, *i* ∉ {*i*_1_, …, *i*_*d*_}, for some subset of (at most) *s* variables, treating the remaining (at least) *d* variables as coefficients of the system. If a parameterization found in this way exists but is not positive, another set of variables should be tried out. This can be systematically addressed by trying out all possible subsets of variables. It requires computation and analysis of at most (nd) parameterizations. Alternatively, one can compute the circuits of degree one of the matroid associated with the equilibrium equations [51].

In some cases, the network structure implies that a positive parameterization of the set of equilibria exists. A set, say {*X*_*k*+1_, …, *X*_*n*_} with *n* − *k* elements for some *k*, is *non-interacting* if two species never appear on the same side of a reaction and they have coefficient at most one in all reactions. In this case the equilibrium equations *f*_*k*+1_(*x*) = ⋯ = *f*_*n*_(*x*) = 0 form a linear system in the variables {*x*_*k*+1_, …, *x*_*n*_}. Provided that the determinant of the coefficient matrix of the linear system is not identically zero, this system can be solved and we obtain a positive parameterization of the non-interacting variables *x*_*k*+1_, …, *x*_*n*_ at equilibrium in terms of the remaining variables *x*_1_, …, *x*_*k*_ [52, 53]. A necessary condition for the determinant of the coefficient matrix not being identically zero is that there is no conservation relation of the form *x*_*i*_1__ + ⋯ + *x*_*i*_*l*__ with *i*_1_, …, *i*_*l*_ ∈ {*k* + 1, …, *n*}. If a non-interacting set with *k* = *d* exists, that is, with *s* = *n* − *d* elements, then this guarantees the existence of the desired parameterization. In the running example there is not a non-interacting set because both species have coefficient 2 in the reaction 2*X*_1_ ⟶ 2*X*_2_.

The non-interacting condition can be relaxed in some cases by requiring that none of the species in {*X*_*k*+1_, …, *X*_*n*_} appear together in a reactant (these sets are called *reactant-non-interacting* [54]). Proceeding as above, provided that the determinant of the coefficient matrix is not identically zero, *x*_*k*+1_, …, *x*_*n*_ can be expressed at equilibrium in terms of *x*_1_, …, *x*_*k*_. Conditions that ensure this is a positive parameterization are given in [54]. In the running example, species *X*_1_ is a reactant-non-interacting set and we can obtain a positive parameterization of *x*_1_ in terms of *x*_2_, see Figs 1 and 2.

If the network admits so-called *toric steady states*, then a positive parameterization also exists [14].

***Step 7: the sign of*** $a(\hat{x})$ ***and the Newton polytope.*** This is perhaps the hardest step of all. We write $a\left(\hat{x}\right)=p\left(\hat{x}\right)/q\left(\hat{x}\right)$ with $q(\hat{x})$ positive for all $\hat{x}$ and would like to determine the sign of $p(\hat{x})$. We first look for conditions that ensure uniqueness of positive equilibria by imposing that all coefficients of $p(\hat{x})$ as a polynomial in $\hat{x}$ have sign (−1)^*s*^.

We next identify the monomials of $p(\hat{x})$, where the sign of the coefficient, say *β*, is (−1)^*s*+1^ for some parameter values. For each of these monomials we check whether the monomial can “dominate” the sign of $p(\hat{x})$. That is to say, if sign(*β*) = (−1)^*s*+1^, then we determine whether there is an $\hat{x}$ such that $p(\hat{x})$ also has the sign (−1)^*s*+1^. If it is the case, then the condition sign(*β*) = (−1)^*s*+1^ is a sufficient condition for multiple equilibria according to Corollary 2(B).

Given a coefficient of a monomial with sign (−1)^*s*+1^, it might not be straightforward to decide if the polynomial $p(\hat{x})$ has the same sign for some value of $\hat{x}$. (For example, the polynomial *x*^2^ − 2*xy* + *y*^2^ = (*x* − *y*)^2^ has one monomial with negative sign, but the polynomial itself can never be negative.) When the number of variables is small, one can attempt to decide the sign as we did in the examples above. Otherwise, our strategy is to determine whether the monomial of interest corresponds to a vertex of the *Newton polytope*. If that is the case, then the monomial can dominate the sign of $p(\hat{x})$ (see §5.2 in the S1 File). The Newton polytope of $p(\hat{x})$ is defined as the convex hull of the exponent vectors $\alpha =\left(\alpha_{1},\cdots ,\alpha_{m}\right)\in R^{m}$ corresponding to the monomials ${\hat{x}}_{1}^{\alpha_{1}}\cdot \ldots \cdot {\hat{x}}_{m}^{\alpha_{m}}$ of $p(\hat{x})$. If *α* is a vertex of the Newton polytope, then there exists $\hat{x}\in R_{>0}^{m}$ such that the sign of $p(\hat{x})$ agrees with the sign of the coefficient of the monomial (see §5.2 in the S1 File).

**An algorithm.** In the previous subsection we have outlined computational criteria that might be used to verify the conditions of the steps in the procedure. These computational criteria are only sufficient, that is, even if they fail the procedure might still work on the given network. For example, a sufficient computational criterion for the absence of boundary equilibria is based on Proposition 2. However, it might happen that Proposition 2 cannot be applied, but that the network nonetheless has no boundary equilibria in stoichiometric compatibility classes with non-empty interior.

**Fig 3:**
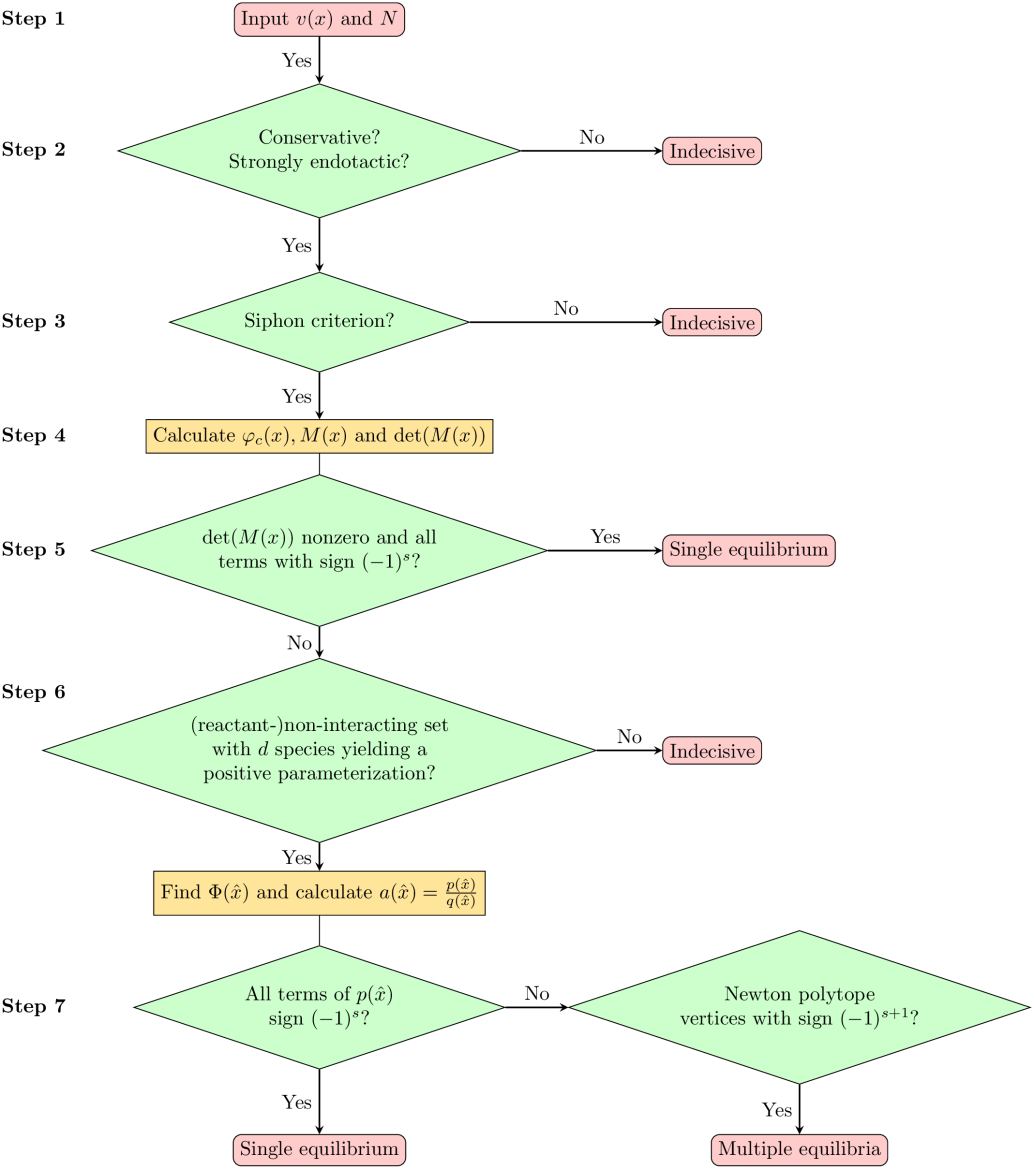
Decision diagram of the algorithm. At each step either the condition is fulfilled or the algorithm terminates indecisively. If that is the case, the corresponding condition might still be verified manually and the algorithm resumed from the next following step.

We have collected sufficient computational criteria that guarantee the conditions of the procedure are fulfilled. In this way the procedure is formulated as an algorithm with decision diagram shown in Fig 3. If one step of the algorithm fails, then we say that the algorithm ends indecisively. In that case we might check whether the step can be verified by other means.

For simplicity, we have restricted to mass-action kinetics. Under this assumption, det(*M*(*x*)) is a polynomial in *x* and the parameters *κ*, and $a(\hat{x})$ is a rational function in $\hat{x}$ and *κ* because the parameterization is assumed to be algebraic.

### Applications to selected examples

To illustrate several aspects of the algorithm we provide a detailed step-by-step analysis of a collection of examples.

#### Two-component system

We have chosen this example to illustrate the situation where an algebraic parameterization is not required, as already det(*M*(*x*)) is of constant sign. The algorithm therefore stops successfully at Step 5 (and consequently skips Step 6 and 7).

We consider a simple version of a two-component system consisting of a histidine kinase HK that autophosphorylates and transfers the phosphate group to a response regulator RR, which undergoes autodephosphorylation. The reactions of the network are

$$\begin{array}{c} \text{HK}\overset{\kappa_{1}}{⟶}{\text{HK}}_{p}\;{\text{HK}}_{p}+\text{RR}\overset{\kappa_{2}}{⟶}\text{HK}+{\text{RR}}_{p}\;{\text{RR}}_{p}\overset{\kappa_{3}}{⟶}\text{RR}. \end{array}$$

We let *X*_1_ = HK, *X*_2_ = HK_*p*_, *X*_3_ = RR and *X*_4_ = RR_*p*_. The stoichiometric matrix *N* and a row reduced matrix *W* such that *WN* = 0 are

$$\begin{array}{c} N=\left(\begin{array}{ccc} -1 & 1 & 0\\ 1 & -1 & 0\\ 0 & -1 & 1\\ 0 & 1 & -1 \end{array}\right),\;W=(\begin{array}{cccc} 1 & 1 & 0 & 0\\ 0 & 0 & 1 & 1 \end{array}). \end{array}$$

The matrix *W* gives rise to the conservation relations *x*_1_ + *x*_2_ = *c*_1_ and *x*_3_ + *x*_4_ = *c*_2_. With mass-action kinetics, the vector of reaction rates is *v*(*x*) = (*κ*_1_*x*_1_, *κ*_2_*x*_2_*x*_3_, *κ*_3_*x*_4_), and the function *f*(*x*) = *Nv*(*x*) is

$$\begin{array}{c} f\left(x\right)=\left(-\kappa_{1}x_{1}+\kappa_{2}x_{2}x_{3},\kappa_{1}x_{1}-\kappa_{2}x_{2}x_{3}-\kappa_{2}x_{2}x_{3}+\kappa_{3}x_{4},\kappa_{2}x_{2}x_{3}-\kappa_{3}x_{4}\right). \end{array}$$

We apply the algorithm to this network.

**Step 1.** Mass-action kinetics fulfills assumption in Eq (2) on the vanishing of reaction rate functions. The function *f*(*x*) and *W* are given above. The matrix *W* is row reduced.

**Step 2.** The network is conservative since (1, 1, 1, 1) ∈ im(*N*)^⊥^. Therefore the network is dissipative.

**Step 3.** The minimal siphons of the network are {*X*_1_, *X*_2_} and {*X*_3_, *X*_4_}. These two sets are the supports of the conservation relations. By Proposition 2, there are no boundary equilibria in any $P_{c}$ as long as $P_{c}^{+}\neq \emptyset$.

**Step 4.** With our choice of *W*, we have *i*_1_ = 1, *i*_2_ = 3. Hence *φ*_*c*_ is obtained by replacing the components *f*_1_(*x*), *f*_3_(*x*) of *f*(*x*) by the expressions derived from the two conservation relations:

$$\begin{array}{c} \varphi_{c}\left(x\right)=\left(x_{1}+x_{2}-c_{1},\kappa_{1}x_{1}-\kappa_{2}x_{2}x_{3},x_{3}+x_{4}-c_{2},\kappa_{2}x_{2}x_{3}-\kappa_{3}x_{4}\right). \end{array}$$

The Jacobian *M*(*x*) of *φ*_*c*_ and its determinant are

$$\begin{array}{ccc} M(x) & = & (\begin{array}{cccc} 1 & 1 & 0 & 0\\ \kappa_{1} & -\kappa_{2}x_{3} & -\kappa_{2}x_{2} & 0\\ 0 & 0 & 1 & 1\\ 0 & \kappa_{2}x_{3} & \kappa_{2}x_{2} & -\kappa_{3} \end{array}).\\ \text{det}(M(x)) & = & \kappa_{1}\kappa_{2}x_{2}+\kappa_{2}\kappa_{3}x_{3}+\kappa_{1}\kappa_{3}, \end{array}$$

**Step 5.** All terms of det(*M*(*x*)) have sign +1 = (−1)^*s*^, since *s* = 2, and thus the conclusion of Corollary 1 holds. The network admits exactly one non-degenerate equilibrium point in every stoichiometric compatibility class with non-empty positive part.

#### Hybrid histidine kinase

This example has been analysed in [55]. Taken with mass-action kinetics the network is known to be multistationary for specific choices of reaction rate constants. We have chosen this example to illustrate how the algorithm can be used to sharpen known results: not only does it establish multistationarity for some parameter values, it provides precise conditions for when it occurs and allows a complete partition of the parameter space into regions with and without multistationarity. It also illustrates the use of an algebraic parameterization, which can be obtained by identifying sets of reactant-non-interacting species, and the use of the Newton polytope in Step 7.

**Fig 4:**
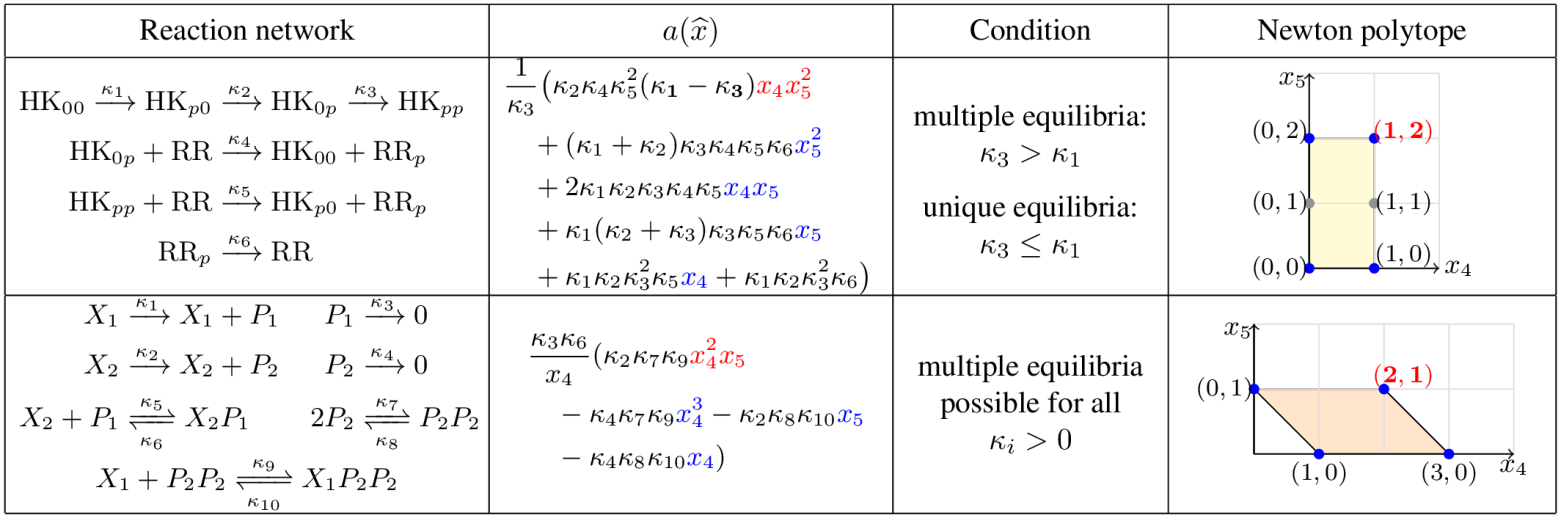
Two examples describing a hybrid histidine kinase (row 1) and a gene transcription network (row 2). *Column 1*: the reaction network; *Column 2*: the function $a(\hat{x})$ where monomials with coefficients of constant sign (−1)^*s*^ are in blue, and those that can have sign (−1)^*s*+1^ are in red; *Column 3*: parameter conditions for multistationarity; *Column 4*: Newton polytope where each point corresponds to the exponent vector of a monomial of the numerator of $a(\hat{x})$ (e.g. (1, 2) is the exponent vector of the monomial $x_{4}x_{5}^{2}$), blue points are the vertices of the Newton polytope and red numbers indicate the exponents of the red monomials in column 2.

This reaction network is an extension of the two-component system discussed above and it is given in the first row of Fig 4. Specifically, the histidine kinase is assumed to be *hybrid*, that is, it has two ordered phosphorylation sites [55]. Whenever the second phosphorylation site is occupied, the phosphate group can be transferred to a response protein.

Using the notation *X*_1_ = HK_00_, *X*_2_ = HK_*p*0_, *X*_3_ = HK_0*p*_, *X*_4_ = HK_*pp*_, *X*_5_ = RR and *X*_6_ = RR_*p*_, the stoichiometric matrix *N* and a row reduced matrix *W* such that *WN* = 0 are

$$\begin{array}{c} N=\left(\begin{array}{cccccc} -1 & 0 & 0 & 1 & 0 & 0\\ 1 & -1 & 0 & 0 & 1 & 0\\ 0 & 1 & -1 & -1 & 0 & 0\\ 0 & 0 & 1 & 0 & -1 & 0\\ 0 & 0 & 0 & -1 & -1 & 1\\ 0 & 0 & 0 & 1 & 1 & -1 \end{array}\right),\;W=\left(\begin{array}{cccccc} 1 & 1 & 1 & 1 & 0 & 0\\ 0 & 0 & 0 & 0 & 1 & 1 \end{array}\right). \end{array}$$

The matrix *W* gives rise to the conservation relations *x*_1_ + *x*_2_ + *x*_3_ + *x*_4_ = *c*_1_ and *x*_5_ + *x*_6_ = *c*_2_. We assume mass-action kinetics

$$\begin{array}{c} v\left(x\right)=\left(\kappa_{1}x_{1},\kappa_{2}x_{2},\kappa_{3}x_{3},\kappa_{4}x_{3}x_{5},\kappa_{5}x_{4}x_{5},\kappa_{6}x_{6}\right), \end{array}$$

and the function is

$$\begin{array}{ccc} f(x) & = & (-\kappa_{1}x_{1}+\kappa_{4}x_{3}x_{5},\kappa_{1}x_{1}-\kappa_{2}x_{2}+\kappa_{5}x_{4}x_{5},-\kappa_{3}x_{3}+\kappa_{2}x_{2}-\kappa_{4}x_{3}x_{5},\\ & & \kappa_{3}x_{3}-\kappa_{5}x_{4}x_{5},-\kappa_{4}x_{3}x_{5}-\kappa_{5}x_{4}x_{5}+\kappa_{6}x_{6},\kappa_{4}x_{3}x_{5}-\kappa_{6}x_{6}+\kappa_{5}x_{4}x_{5}). \end{array}$$

We apply the algorithm to this network.

**Step 1.** Mass-action kinetics fulfills assumption in Eq (2) on the vanishing of reaction rate functions. The function *f*(*x*) and *W* are given above. The matrix *W* is row reduced.

**Step 2.** Since (1, 1, 1, 1, 1, 1) ∈ im(*N*)^⊥^ the network is conservative and hence dissipative.

**Step 3.** The network has two minimal siphons {*X*_1_, *X*_2_, *X*_3_, *X*_4_} and {*X*_5_, *X*_6_}, which are respectively the supports of the two conservation relations. We apply Proposition 2 to conclude that there are no boundary equilibria in any $P_{c}$ as long as $P_{c}^{+}\neq \emptyset$.

**Step 4.** Since *i*_1_ = 1, *i*_2_ = 5, the function *φ*_*c*_ is obtained by replacing the components *f*_1_(*x*), *f*_5_(*x*) of *f*(*x*) by the expressions derived from the two conservation relations:

$$\begin{array}{ccc} \varphi_{c}\left(x\right) & = & (x_{1}+x_{2}+x_{3}+x_{4}-c_{1},\kappa_{1}x_{1}-\kappa_{2}x_{2}+\kappa_{5}x_{4}x_{5},-\kappa_{3}x_{3}+\kappa_{2}x_{2}-\kappa_{4}x_{3}x_{5},\\ & & \kappa_{3}x_{3}-\kappa_{5}x_{4}x_{5},x_{5}+x_{6}-c_{2},\kappa_{4}x_{3}x_{5}-\kappa_{6}x_{6}+\kappa_{5}x_{4}x_{5}). \end{array}$$

The Jacobian *M*(*x*) of *φ*_*c*_(*x*) and its determinant are

$$\begin{array}{ccc} M(x) & = & (\begin{array}{cccccc} 1 & 1 & 1 & 1 & 0 & 0\\ \kappa_{1} & -\kappa_{2} & 0 & \kappa_{5}x_{5} & \kappa_{5}x_{4} & 0\\ 0 & \kappa_{2} & -\kappa_{3}-\kappa_{4}x_{5} & 0 & -\kappa_{4}x_{3} & 0\\ 0 & 0 & \kappa_{3} & -\kappa_{5}x_{5} & -\kappa_{5}x_{4} & 0\\ 0 & 0 & 0 & 0 & 1 & 1\\ 0 & 0 & \kappa_{4}x_{5} & \kappa_{5}x_{5} & \kappa_{4}x_{3}+\kappa_{5}x_{4} & -\kappa_{6} \end{array}),\\ \text{det}(M(x)) & = & \kappa_{2}\kappa_{4}\kappa_{5}\left(\kappa_{1}-\kappa_{3}\right)x_{3}x_{5}+\kappa_{1}\kappa_{2}\kappa_{4}\kappa_{5}x_{4}x_{5}+\kappa_{4}\kappa_{5}\kappa_{6}\left(\kappa_{1}+\kappa_{2}\right)x_{5}^{2}\\ & & +\kappa_{1}\kappa_{2}\kappa_{3}\kappa_{4}x_{3}+\kappa_{1}\kappa_{2}\kappa_{3}\kappa_{5}x_{4}+\kappa_{1}\kappa_{5}\kappa_{6}\left(\kappa_{3}+\kappa_{2}\right)x_{5}+\kappa_{1}\kappa_{2}\kappa_{3}\kappa_{6}. \end{array}$$

**Step 5.** The sign of the first coefficient of det(*M*(*x*)) depends on the parameters. If *κ*_1_ ≥ *κ*_3_, then the sign is positive and det(*M*(*x*)) has sign +1 = (−1)^4^ (*s* = 4) as the remaining terms are positive. According to Corollary 1, there is a single non-degenerate equilibrium in each stoichiometric compatibility class with non-empty positive part. If *κ*_1_ < *κ*_3_, then Corollary 1 cannot be applied. We proceed to the next step to investigate the parameter space further.

**Step 6.** The set {*X*_1_, *X*_2_, *X*_3_, *X*_6_} is reactant-non-interacting and consists of *s* = 4 elements. We solve the equilibrium equations *f*_1_ = *f*_2_ = *f*_3_ = *f*_6_ = 0 for *x*_1_, *x*_2_, *x*_3_, *x*_6_. This gives the following algebraic parameterization $\Phi :R_{>0}^{2}⟶V\cap R_{>0}^{6}$ of the set of equilibria in terms of $\hat{x}=\left(x_{4},x_{5}\right)$:

$$\begin{array}{ccc} \Phi (x_{4},x_{5}) & = & (\frac{\kappa_{4}\kappa_{5}x_{4}x_{5}^{2}}{\kappa_{1}\kappa_{3}},\frac{\kappa_{5}\left(\kappa_{4}x_{5}+\kappa_{3}\right)x_{4}x_{5}}{\kappa_{2}\kappa_{3}},\frac{\kappa_{5}x_{4}x_{5}}{\kappa_{3}},x_{4},x_{5},\frac{\kappa_{5}\left(\kappa_{4}x_{5}+\kappa_{3}\right)x_{4}x_{5}}{\kappa_{3}\kappa_{6}}). \end{array}$$

The function $a(\hat{x})$, which is det(*M*(*x*)) evaluated at Φ(*x*_4_, *x*_5_), is the polynomial given in the first row of Fig 4.

**Step 7.** We assume *κ*_1_ < *κ*_3_, as the case *κ*_1_ ≥ *κ*_3_ is analysed in Step 5. Only one coefficient of $a(\hat{x})$ has sign −1 = (−1)^*s*+1^ = (−1)^5^. The monomial associated with this term is $x_{4}x_{5}^{2}$. As the point (1, 2) (the degrees of the monomial) is a vertex of the Newton polytope (see Fig 4), then there exists $\hat{x}\in R_{>0}^{m}$ such that the sign of $a(\hat{x})$ is −1. Corollary 2(B) implies that there exists *c* = (*c*_1_, *c*_2_) such that $P_{c}$ contains at least two positive equilibria.

Multistationarity is thus completely characterized by the inequality *κ*_3_ > *κ*_1_. This condition states that the reaction rate constant for phosphorylation of the first site of the hybrid kinase is larger if the second site is phosphorylated than if it is not.

#### Gene transcription network

We consider the gene transcription network given in row 2 of Fig 4. This example has been studied in [56]. The particularities of this example are that the network is dissipative but not conservative, and that it displays multistationarity for *all* parameters *κ*. Further, this network illustrates the situation where the algorithm stops inconclusively at some step, but can be resumed after successful manual verification.

The network represents a gene transcription motif with two proteins *P*_1_, *P*_2_, produced by their respective genes *X*_1_, *X*_2_, and such that *P*_2_ dimerises [56]. Further, the proteins cross regulate each other as depicted in Fig 4. Using the notation *X*_1_ = *X*_1_, *X*_2_ = *X*_2_, *X*_3_ = *P*_1_, *X*_4_ = *P*_2_, *X*_5_ = *X*_2_*P*_1_, *X*_6_ = *P*_2_*P*_2_, and *X*_7_ = *X*_1_*P*_2_*P*_2_ the stoichiometric matrix *N* and a row reduced matrix *W* such that *WN* = 0 are

$$\begin{array}{ccc} N & = & (\begin{array}{cccccccccc} 0 & 0 & 0 & 0 & 0 & 0 & 0 & 0 & -1 & 1\\ 0 & 0 & 0 & 0 & -1 & 1 & 0 & 0 & 0 & 0\\ 1 & 0 & -1 & 0 & -1 & 1 & 0 & 0 & 0 & 0\\ 0 & 1 & 0 & -1 & 0 & 0 & -2 & 2 & 0 & 0\\ 0 & 0 & 0 & 0 & 1 & -1 & 0 & 0 & 0 & 0\\ 0 & 0 & 0 & 0 & 0 & 0 & 1 & -1 & -1 & 1\\ 0 & 0 & 0 & 0 & 0 & 0 & 0 & 0 & 1 & -1 \end{array}),\\ W & = & (\begin{array}{ccccccc} 1 & 0 & 0 & 0 & 0 & 0 & 1\\ 0 & 1 & 0 & 0 & 1 & 0 & 0 \end{array}). \end{array}$$

From *W* we find the conservation relations *x*_1_ + *x*_7_ = *c*_1_ and *x*_2_ + *x*_5_ = *c*_2_. Here *s* = 5. We consider mass-action kinetics such that

$$\begin{array}{c} v\left(x\right)=\left(\kappa_{1}x_{1},\kappa_{2}x_{2},\kappa_{3}x_{3},\kappa_{4}x_{4},\kappa_{5}x_{2}x_{3},\kappa_{6}x_{5},\kappa_{7}x_{4}^{2},\kappa_{8}x_{6},\kappa_{9}x_{1}x_{6},\kappa_{10}x_{7}\right) \end{array}$$

and *f*(*x*) = *Nv*(*x*) is the function

$$\begin{array}{ccc} & & (-\kappa_{9}x_{1}x_{6}+\kappa_{10}x_{7},-\kappa_{5}x_{2}x_{3}+\kappa_{6}x_{5},\kappa_{1}x_{1}-\kappa_{3}x_{3}-\kappa_{5}x_{2}x_{3}+\kappa_{6}x_{5},\kappa_{2}x_{2}-\kappa_{4}x_{4}\\ & & -2\kappa_{7}x_{4}^{2}+2\kappa_{8}x_{6},\kappa_{5}x_{2}x_{3}-\kappa_{6}x_{5},\kappa_{7}x_{4}^{2}-\kappa_{8}x_{6}-\kappa_{9}x_{1}x_{6}+\kappa_{10}x_{7},\kappa_{9}x_{1}x_{6}-\kappa_{10}x_{7}). \end{array}$$

We apply the algorithm to this network:

**Step 1.** Mass-action kinetics fulfills assumption in Eq (2) on the vanishing of reaction rate functions. The function *f*(*x*) and *W* are given above. The matrix *W* is row reduced.

**Step 2.** The network is neither conservative nor strongly endotactic. Thus the algorithm terminates inconclusive. We take a manual approach: we pick $\omega_{c}=\left(1,1,1,1,2,2,3\right)\in R_{>0}^{7}$ and observe

$$\begin{array}{c} \omega_{c}\cdot f\left(x\right)=\kappa_{1}x_{1}+\kappa_{2}x_{2}-\kappa_{3}x_{3}-\kappa_{4}x_{4}. \end{array}$$

Note that *x*_1_, *x*_2_ are bounded (due to the conservation relations) while *x*_3_, *x*_4_ can be arbitrarily large. Then, for *x*_3_, *x*_4_ large enough, *ω*_*c*_ ⋅ *f*(*x*) < 0 and the network is dissipative by Proposition 1 (as has been shown in [56] by other means).

**Step 3.** This network has two minimal siphons: {*X*_1_, *X*_7_} and {*X*_2_, *X*_5_}, which are the supports of the two conservation relations. Therefore, by Proposition 2, there are no boundary steady states in stoichiometric compatibility class with non-empty positive part.

In section §5.1 in the S1 File we illustrate how to apply a simplification technique, based on the removal of so-called *intermediates* and *catalysts*, to check whether Proposition 2 holds for this network.

**Step 4.** Using that *i*_1_ = 1, *i*_2_ = 2 for our choice of *W*, the function *φ*_*c*_(*x*) is:

$$\begin{array}{ccc} & & (x_{1}+x_{7}-c_{1},x_{2}+x_{5}-c_{2},\kappa_{1}x_{1}-\kappa_{3}x_{3},-\kappa_{5}x_{2}x_{3}+\kappa_{6}x_{5}\kappa_{2}x_{2}-\kappa_{4}x_{4}\\ & & -2\kappa_{7}x_{4}^{2}+2\kappa_{8}x_{6},\kappa_{5}x_{2}x_{3}-\kappa_{6}x_{5},\kappa_{7}x_{4}^{2}-\kappa_{8}x_{6}-\kappa_{9}x_{1}x_{6}+\kappa_{10}x_{7},\kappa_{9}x_{1}x_{6}-\kappa_{10}x_{7}). \end{array}$$

The matrix *M*(*x*) and its determinant are:

$$\begin{array}{ccc} M(x) & = & (\begin{array}{ccccccc} 1 & 0 & 0 & 0 & 0 & 0 & 1\\ 0 & 1 & 0 & 0 & 1 & 0 & 0\\ \kappa_{1} & -\kappa_{5}x_{3} & -\kappa_{5}x_{2}-\kappa_{3} & 0 & \kappa_{6} & 0 & 0\\ 0 & \kappa_{2} & 0 & -4\kappa_{7}x_{4}-\kappa_{4} & 0 & 2\kappa_{8} & 0\\ 0 & \kappa_{5}x_{3} & \kappa_{5}x_{2} & 0 & -\kappa_{6} & 0 & 0\\ -\kappa_{9}x_{6} & 0 & 0 & 2\;\kappa_{7}x_{4} & 0 & -\kappa_{9}x_{1}-\kappa_{8} & \kappa_{10}\\ \kappa_{9}x_{6} & 0 & 0 & 0 & 0 & \kappa_{9}x_{1} & -\kappa_{10} \end{array}),\\ \text{det}(M(x)) & = & 2\kappa_{1}\kappa_{2}\kappa_{5}\kappa_{7}\kappa_{9}x_{1}x_{2}x_{4}-\kappa_{3}\kappa_{4}\kappa_{5}\kappa_{8}\kappa_{9}x_{3}x_{6}-\kappa_{3}\kappa_{4}\kappa_{5}\kappa_{8}\kappa_{10}x_{3}\\ & & -\kappa_{3}\kappa_{4}\kappa_{6}\kappa_{8}\kappa_{9}x_{6}-\kappa_{3}\kappa_{4}\kappa_{6}\kappa_{8}\kappa_{10}. \end{array}$$

**Step 5.** One coefficient of det(*M*(*x*)) has sign (−1)^*s*+1^ = 1 for all values of *κ*. Thus we proceed to the next step.

**Step 6.** There is not a set of non-interacting species nor reactant-non-interacting with *s* = 5 elements. Thus the algorithm terminates inconclusively.

We take a manual approach and solve the equilibrium equations *f*_3_ = *f*_4_ = *f*_5_ = *f*_6_ = *f*_7_ = 0 for *x*_1_, *x*_2_, *x*_3_, *x*_6_, *x*_7_. This gives the following algebraic parameterization $\Phi :R_{>0}^{2}⟶R_{>0}^{7}$ of the set of equilibria in terms of $\hat{x}=\left(x_{4},x_{5}\right)$:

$$\begin{array}{c} \Phi \left(x_{4},x_{5}\right)=\left(\frac{\kappa_{2}\kappa_{3}\kappa_{6}x_{5}}{\kappa_{1}\kappa_{4}\kappa_{5}x_{4}},\frac{\kappa_{4}x_{4}}{\kappa_{2}},\frac{\kappa_{2}\kappa_{6}x_{5}}{\kappa_{4}\kappa_{5}x_{4}},x_{4},x_{5},\frac{\kappa_{7}x_{4}^{2}}{\kappa_{8}},\frac{\kappa_{2}\kappa_{3}\kappa_{6}\kappa_{7}\kappa_{9}x_{4}x_{5}}{\kappa_{1}\kappa_{4}\kappa_{5}\kappa_{8}\kappa_{10}}\right). \end{array}$$

Evaluating det(*M*(*x*)) at Φ(*x*_4_, *x*_5_) we obtain the polynomial

$$\begin{array}{c} a\left(x_{4},x_{5}\right)=\frac{\kappa_{3}\kappa_{6}}{x_{4}}\left(\kappa_{2}\kappa_{7}\kappa_{9}x_{4}^{2}x_{5}-\kappa_{4}\kappa_{7}\kappa_{9}x_{4}^{3}-\kappa_{2}\kappa_{8}\kappa_{10}x_{5}-\kappa_{4}\kappa_{8}\kappa_{10}x_{4}\right). \end{array}$$

**Step 7.** The coefficient of the monomial $x_{4}^{2}x_{5}$ of the numerator of *a*(*x*_4_, *x*_5_) has sign (−1)^*s*+1^ = (−1)^6^ = 1. Since the monomial $x_{4}^{2}x_{5}$ is a vertex of the associated Newton polytope (see Fig 4), there exists $\left(x_{4},x_{5}\right)\in R_{>0}^{2}$ such that the sign of *a*(*x*_4_, *x*_5_) is 1. We conclude from Corollary 2(B) that for all *κ*_*i*_ > 0 there exists *c* such that $P_{c}$ contains at least two positive equilibria.

#### Special classes of networks

There are several classes of networks for which some of the steps of the procedure are automatically fulfilled. We review some of them here.

Post-Translational Modification (PTM) networks consist of enzymes (*E*_*i*_), substrates (*S*_*i*_) and intermediate species (*Y*_*i*_) [28]. Allowed reactions are of the form

$$\begin{array}{c} E_{i}+S_{j}⟶Y_{k},\;Y_{k}⟶E_{i}+S_{j},\;Y_{j}⟶Y_{i}. \end{array}$$

All intermediates are assumed to be the reactant, respectively, the product of some reaction. These networks are conservative (hence dissipative) and boundary equilibria are precluded provided the underlying substrate network obtained by ignoring enzymes and intermediates is strongly connected [50], see also §5.1 in the S1 File. When equipped with mass-action kinetics, these networks have a non-interacting set with *d* elements consisting of all enzymes and some of the substrates, namely one per (minimal) conservation relation involving the substrates [28]. Thus, a positive parameterization can always be found under the conditions stated above in Step 6. The class of PTM networks is contained in the class of cascades of PTM networks. Also this class admits a positive parameterization in terms of the concentrations of the enzymes and some of the substrate forms [29].

Cascades of PTM networks might further be generalized to so-called MESSI networks [57]. These networks are all conservative. Easy-to-check conditions for the absence of boundary equilibria and to decide whether the network admits toric steady states (and hence a positive parameterization) are given in [57].

A class of networks that cannot have boundary equilibria in any stoichiometric compatibility class with non-empty interior is given in [58].

The two examples in Table 1 are both PTM networks. Hence they are conservative and positive parameterizations exist. The underlying substrate network is strongly connected (they pass the criterion based on minimal siphons). For both networks the conditions shown in Table 1 are obtained by the algorithm. See §6.1 and §6.2 in the S1 File. For illustration purposes, we apply the algorithm in §6.3 of the S1 File to an additional network and show that it is monostationary.

### Computational issues

The computational complexity of some of the steps in the procedure are demanding. Some conditions can be checked using linear algebra and do not depend on parameter values, others depend on parameter values and require symbolic manipulations. In some situations, the calculation can be done for even large networks at the cost of time, while in other situations symbolic software (like Mathematica and Maple) have inherent limits to what it can process. We offer here a few remarks about computational strategies and time complexity.

1. Dissipativity. There are efficient algorithms to check whether the network is conservative and strongly endotactic, using linear algebra or mixed-integer linear programming [21, 47]. We are not aware of a systematic way to check if Proposition 1 is fulfilled or not.
2. Finding the minimal siphons of a network requires in general exponential time and there might be exponentially many of these [59]. Different algorithms developed in Petri Net theory can be applied to find the minimal siphons; see for example [48, 49, 59] and references therein. The complexity of this computation can often be substantially reduced by removing so-called intermediates and catalysts from the network [50] (see §5.1 in the S1 File for details).
3. Finding all non-interacting and reactant-non-interacting sets requires in general exponential time. One strategy is the following. We first remove all species *S*_*i*_ for which *α*_*ij*_ > 1 or *β*_*ij*_ > 1 for some reaction *R*_*j*_ (the latter constraint is omitted if we are looking for reactant-non-interacting sets only). Then we build non-interacting (reactant-non-interacting) sets by adding new species recursively until no more species can be added without having an interacting pair of species in the set.
4. Calculation of the symbolic determinant of the matrix *M*(*x*), and hence also of $a(\hat{x})$, often fails in our experience for networks with more than 20 variables on common laptops [60]. However, this clearly depends on the sparsity of the matrix *M*(*x*), that is, on the number and order of the reactions. Strategies to reduce the complexity of the computation by expanding the determinant along the non-symbolic rows (conservation relations) were inspected in [60]. Specialized software like Singular [61] and/or better hardware could probably push what is possible to something closer to 50 variables. At this size, however, what might best be called ‘cognitive limitations’ come into play: symbolic software typically has problems with collecting and simplifying terms ‘the right way’ if there are many variables and/or parameters. And if terms are not collected appropriately it might be difficult, if not impossible, to decide on the sign of the polynomial coefficients. Our approach is therefore best suited to systems of moderate size (say 20-30 variables). Furthermore, it is our experience that large non-linear models tend to be multistationary because of the many non-linear dependencies that typically are present [60].
5. Positive parameterizations: The worst case scenario involves checking ∑i=1d(ni) different sets of variables, each with at most *d* variables.
6. Finding the vertices of the Newton polytope can be done with existing symbolic software, for example Polymake [62] or Maple, as we demonstrate in the S1 File.

We stress that it is always beneficial to guide the procedure/algorithm whenever possible in the sense that, if something is known for the network, there is no reason to go through many possibilities.

## Discussion

The main result of this paper, the procedure to identify parameter regions for unique and multiple equilibria, combines Brouwer degree theory and algebraic geometry. In particular, under the assumptions of Corollary 2, we show that there exist stoichiometric compatibility classes with at least two equilibria if, and only if, a certain multivariate polynomial can attain a specific sign.

Discriminating regions of the parameter space where multistationarity occurs is a hard mathematical problem, theoretically addressable by computationally expensive means [25]. Our approach beautifully overcomes these difficulties by building on a simple idea, the computation of the Brouwer degree of a function related to a dissipative network. Additionally, not only closed-form expressions in the parameters are obtained, but, as illustrated in examples, these expressions are often interpretable in biochemical terms, providing an explanation of *why* multistationarity occurs.

The procedure applies theoretically to any choice of algebraic reaction rate functions. However, in practice, the procedure works well with mass-action kinetics. For example, we have considered the two-site phosphorylation cycle depicted in the second row of Table 1, but now modelled with Michaelis-Menten kinetics instead of mass-action kinetics. This network is known to be multistationary [63], and the conditions to apply Corollary 1 and Corollary 2 are valid. However, a positive algebraic parameterization does not exist, and hence our approach cannot be used to find parameter conditions for multistationarity.

However, Corollary 1 might be used with rational reaction rate functions for monostationary networks. This is the case for example for the one-site phosphorylation cycle $S⇌S_{p}$ with Michaelis-Menten kinetics [63]. This network has two species and rank one. The sign of det(*M*(*x*)) is −1 for all parameter values and all $x\in R_{>0}^{2}$. By Corollary 1, the network admits exactly one positive equilibrium in every stoichiometric compatibility class $P_{c}$ with $P_{c}^{+}\neq \emptyset$ for all parameter values.

If a reaction network does not have any conservation relation, then the set of equilibria consists typically of a finite number of points. In this case an algebraic parameterization is an algebraic expression of the equilibria in terms of the parameters of the system. Since *m* = 0, then $R^{m}$ consists of a single point and it follows directly that there is a unique equilibrium. Such an expression rarely exists. Therefore the procedure applies mainly to reaction networks with conservation relations. In particular, this rules out reaction networks where each species is produced and degraded.

Several natural questions remain outside the reach of our procedure. Firstly one would like to determine the particular stoichiometric compatibility classes for which there are multiple equilibria. As stated in Corollary 2, if $\text{sign}\left(a\left(\hat{x}\right)\right)={\left(-1\right)}^{s+1}$, then $c:=W\Phi (\hat{x})$ defines a stoichiometric compatibility class with multiple equilibria. However, this only establishes *c* indirectly through $\hat{x}$. In some situations, it might be possible to find a positive parametrization that uses some of the conservation relations (ideally, all but one) and the stoichiometric compatibility classes with multiple/single equilibria would be determined up to a single parameter.

Secondly, one could ask for parameter regions that differentiate between the precise number of equilibria (that is, 0, 1, 2, …). This question should be seen in conjunction with the previous question: in typical examples, when there are two equilibria in a particular stoichiometric compatibility class, then there exists another class for which there are three. Hence the number of equilibria cannot be separated from the stoichiometric compatibility classes.

A third question concerns the stability of the equilibria, which cannot be obtained from our procedure. It is, however, known that if the sign of the Jacobian evaluated at an equilibrium is (−1)^*s*+1^, then it is unstable [34]. This is in particular the case for an equilibrium fulfilling the condition in Corollary 2(B).

We have shown that for some reaction networks our procedure can be formulated as an algorithm. We consider therefore our research a step in the direction of providing ‘black box tools’ to analyse complex dynamical systems. Such tools would easily find their use in systems and synthetic biology, where it is commonplace to consider (many) competing models. A particular problem is to exclude models that cannot explain observed qualitative features, such as multistationarity.

## Methods

We used Maple for the symbolic computations, such as finding det(*M*(*x*)), the positive parameterizations, $a(\hat{x})$ and the vertices of the Newton polytope.

## Supporting information

S1 File Proof of mathematical statements and examples. In this document we first prove the claims of the main text. Next, we provide details on how to check the steps of the procedure. Finally, we give details of the examples in Table 1 and include an extra example which is a PTM network.
(PDF)
