# Supplementary material for: Identifying parameter regions for multistationarity
Source: PLoS Comput Biol. 2017 Oct 3;13(10):e1005751. doi: 10.1371/journal.pcbi.1005751 (PMC5626113; doi:10.1371/journal.pcbi.1005751)
Supplement: S1 File — In this document we first prove the claims of the main text. Next, we provide details on how to check the steps of the procedure. Finally, we give details of the examples in Table 1 and include an extra example which is a PTM network. (PDF) [file pcbi.1005751.s001.pdf]

Identifying parameter regions for multistationarity

Supplementary Information

Carsten Conradi, Elisenda Feliu, Maya Mincheva, Carsten Wiuf

July 22, 2017

In this document we prove the claims of the main text.

Sections 1 to 4 focus on the proofs of the theorem and its corollaries in the main text. We start by introducing some preliminaries before recapitulating the main facts about Brouwer degree theory. Then we compute the Brouwer degree for a special class of functions (Theorem 2.3). We proceed to introduce the necessary background on reaction networks and to state and prove a key result regarding the Brouwer degree of a reaction network with a dissipative semiflow (Theorem 3.5). In Section 4 we use Theorem 3.5 to prove Theorem 1 of the main text. The first four sections of the document are self-contained and do not require parallel reading of the main text. For this reason some parts of the main text are repeated here for convenience.

Subsequently in Section 5, we provide details on how to check the steps in the procedure of the main text. In Section 6 we give details of the examples in the main text and apply the algorithm to an extra network that is monostationary.

Contents

1 Preliminaries **2**
- 1.1 Convex sets 2
- 1.2 Functions 2

2 Brouwer degree and a theorem **3**
- 2.1 Brouwer degree 3
- 2.2 The Brouwer degree for a special class of functions 4

3 Chemical reaction networks **6**
- 3.1 Setting 6
- 3.2 Conservative and dissipative networks 6
- 3.3 Degree for dissipative semiflows 8

4 Multistationarity in dissipative networks **11**

5 Details on the steps of the procedure **14**
- 5.1 On siphons and boundary equilibria 14
- 5.2 Newton polytope 16

6 Details on the examples in the main text **17**
- 6.1 Phosphorylation of two substrates 17
- 6.2 Two-site phosphorylation system 20
- 6.3 Two-substrate enzyme catalysis 24

1 Preliminaries

1.1 Convex sets

We let $\mathbb{R}^n_{\geq 0}$ denote the non-negative orthant of $\mathbb{R}^n$ and $\mathbb{R}^n_{>0}$ denote the positive orthant of $\mathbb{R}^n$.

For a subset B of $\mathbb{R}^n$, we let $\mathrm{bd}(B)$ denote the boundary of B and $\mathrm{cl}(B)$ the closure of B, such that $\mathrm{cl}(B) = \mathrm{bd}(B) \cup B$. If B is open, then $\mathrm{bd}(B) \cap B = \emptyset$. If B is bounded, then $\mathrm{cl}(B)$ is compact.

A set B is *convex* if the following holds:

$$\text{if} \quad x_1, x_2 \in B \quad \text{then} \quad \lambda x_1 + (1-\lambda)x_2 \in B \quad \text{for all} \quad 0 \leq \lambda \leq 1.$$

Let $B \subseteq \mathbb{R}^n$ be a convex set. We say that $v \in \mathbb{R}^n$ *points inwards* B at $x \in \mathrm{bd}(B)$ if $x + \epsilon v \in \mathrm{cl}(B)$ for all $\epsilon > 0$ small enough. In particular, $v = 0$ points inwards B at all $x \in \mathrm{bd}(B)$. If v points inwards B at $x \in \mathrm{bd}(B)$, then it also points inwards $\mathrm{cl}(B)$ at $x \in \mathrm{bd}(B)$. The vector v *points outwards* B at $x \in \mathrm{bd}(B)$, if it does not point inwards B at $x \in \mathrm{bd}(B)$.

We will use the following facts about convex sets.

Lemma 1.1. *Let $B \subseteq \mathbb{R}^n$ be a convex set. Then the following holds:*

(i) The closure $\mathrm{cl}(B)$ *of B is convex.*

(ii) Assume B is open and consider $x_1 \in B$, $x_2 \in \mathrm{bd}(B)$. Let

$$[x_1, x_2) = \{tx_1 + (1-t)x_2 \mid 0 < t \leq 1\}$$

be the half-closed line segment between x_1 and x_2. Then $[x_1, x_2) \in B$.

(iii) Let $x_1 \in B$ and $x_2 \in \mathrm{bd}(B)$. Then the vector $x_1 - x_2$ points inwards B at x_2. If B is open, then the vector $x_2 - x_1$ points outwards B at x_2.

Proof. (i) See Theorem 6.2 in [10]. (ii) See Theorems 6.1 in [10]. (iii) Consider $x = x_2 + \epsilon(x_1 - x_2) = (1-\epsilon)x_2 + \epsilon x_1$ with $0 < \epsilon < 1$. By convexity, x belongs to $\mathrm{cl}(B)$, hence $x_1 - x_2$ points inwards B at $x_2 \in \mathrm{bd}(B)$. Assume that $x_2 - x_1$ also points inwards B at x_2 and that B is open. Then, for small ϵ we have $x = x_2 + \epsilon(x_1 - x_2) \in B$ by (ii) (which is stronger than $x \in \mathrm{cl}(B)$), and $x' = x_2 + \epsilon(x_2 - x_1) \in \mathrm{cl}(B)$ by definition of pointing inwards. Again by (ii), $\frac{1}{2}x + \frac{1}{2}x' = x_2 \in B$, contradicting that $x_2 \in \mathrm{bd}(B)$ (B is open). Hence $x_2 - x_1$ points outwards B at x_2. □

1.2 Functions

Given an open set $B \subseteq \mathbb{R}^n$, we let $\mathcal{C}^k(B, \mathbb{R}^m)$ denote the set of $\mathcal{C}^k$-functions from B to $\mathbb{R}^m$. If B is open and bounded, then we let $\mathcal{C}^k(\mathrm{cl}(B), \mathbb{R}^m)$ denote the subset of $\mathcal{C}^k(B, \mathbb{R}^m)$-functions f whose j-th derivative $d^j f$, $j = 0, \ldots, k$, extends continuously to the boundary of B. Equivalently, $d^j f$ is uniformly continuous in B for $j = 0, \ldots, k$, since $\mathrm{cl}(B)$ is compact.

For $f \in \mathcal{C}^1(B, \mathbb{R}^n)$ and $x^* \in B$, we let $J_f(x^*) \in \mathbb{R}^{n \times n}$ be the *Jacobian* of f evaluated at x^*, that is, $J_f(x^*)$ is the matrix with (i,j)-entry $\partial f_i(x^*)/\partial x_j$. We say that $y \in \mathbb{R}^n$ is a *regular value* for f if $J_f(x)$ is non-singular for all $x \in B$ such that $y = f(x)$. If this is not the case, then we say that y is a *critical value* for f.

If $B \subseteq \mathbb{R}^n$ is open and bounded, $f \in \mathcal{C}^1(\mathrm{cl}(B), \mathbb{R}^n)$ and y is a regular value for f such that $y \notin f(\mathrm{bd}(B))$, then the set

$$\{x \in B | f(x) = y\}.$$

is finite [13, Lemma 1.4].

2 Brouwer degree and a theorem

2.1 Brouwer degree

We first recall basic facts about the *Brouwer degree.* We refer to Section 14.2 in [12] for background and fundamental properties of the Brouwer degree. See also the lecture notes by Vandervorst [13].

In this section we let $B \subseteq \mathbb{R}^n$ be an open bounded set. We use the symbol $\deg(f, B, y)$ to denote the Brouwer degree (which is an integer number) of a function $f \in \mathcal{C}^0(\mathrm{cl}(B), \mathbb{R}^n)$ with respect to (B, y), $y \in \mathbb{R}^n \setminus f(\mathrm{bd}(B))$.

A main property of the Brouwer degree is that if $y \notin f(\mathrm{cl}(B))$, then $\deg(f, B, y) = 0$ (but not vice versa) and if $\deg(f, B, y) \neq 0$, then there exists at least one $x \in B$ such that $y = f(x)$. In particular, the Brouwer degree can be used to study the number of solutions to the equation

$$f(x) = y, \qquad x \in B,$$

provided $y \notin f(\mathrm{bd}(B))$ and $f \in \mathcal{C}^0(\mathrm{cl}(B), \mathbb{R}^n)$.

The Brouwer degree $\deg(f, B, y)$ is characterized by the following properties:

(A1) **Normalization.** Let id_B denote the identity map from B to itself. If $y \in B$, then

$$\deg(\mathrm{id}_B, B, y) = 1.$$

(A2) **Additivity.** If B_1 and B_2 are disjoint open subsets of B such that $y \notin f\big(\mathrm{cl}(B) \setminus (B_1 \cup B_2)\big)$, then

$$\deg(f, B, y) = \deg(f, B_1, y) + \deg(f, B_2, y).$$

(A3) **Homotopy invariance.** Let $f, g\colon \mathrm{cl}(B) \to \mathbb{R}^n$ be two homotopy equivalent $\mathcal{C}^0$-functions via a continuous homotopy $H\colon \mathrm{cl}(B) \times [0,1] \to \mathbb{R}^n$ such that $H(x, 0) = f(x)$ and $H(x, 1) = g(x)$. If $y \notin H(\mathrm{bd}(B) \times [0,1])$, then

$$\deg(f, B, y) = \deg(g, B, y).$$

(A4) **Translation invariance.** $\deg(f, B, y) = \deg(f - y, B, 0)$.

To prove our main result (Theorem 4.1 below) we need the following well-known property of the Brouwer degree, see e.g. [12, Theorem 14.4]:

Theorem 2.1. *Let $f \in \mathcal{C}^1(\mathrm{cl}(B), \mathbb{R}^n)$ with $B \subseteq \mathbb{R}^n$ an open bounded set. If y is a regular value for f and $y \notin f(\mathrm{bd}(B))$, then*

$$\deg(f, B, y) = \sum_{\{x \in B \mid f(x) = y\}} \mathrm{sign}(\det(J_f(x))), \tag{1}$$

where the sum over an empty set is defined to be zero.

Corollary 2.2. *Under the assumptions of Theorem 2.1, assume* $\deg(f, B, y) = \pm 1$. *Then the equation $f(x) = y$ has at least one solution $x \in B$ and the number of solutions in B is odd.*

2.2 The Brouwer degree for a special class of functions

In this section we use Theorem 2.1 and the homotopy invariance of the Brouwer degree (A3) to compute the Brouwer degree of certain functions. Specifically, we are concerned with $\mathcal{C}^1$-functions

$$f\colon \mathbb{R}^n_{\geq 0} \to \mathbb{R}^n, \tag{2}$$

and matrices $W \in \mathbb{R}^{d\times n}$ of maximal rank d. A priori there is no relationship between f and W.

Assume that W is row reduced and let $i_1, \dots, i_d$ be the indices of the first non-zero coordinate of each row, $i_1 < \dots < i_d$. Let $c \in \mathbb{R}^d$ and define the $\mathcal{C}^1$-function

$$\varphi_c\colon \mathbb{R}^n_{\geq 0} \to \mathbb{R}^n$$

by

$$\varphi_c(x)_i = \begin{cases} f_i(x) & i \notin \{i_1, \dots, i_d\} \\ (Wx - c)_i & i \in \{i_1, \dots, i_d\}. \end{cases} \tag{3}$$

We say that φ_c is constructed from f and W. The dependence of φ_c on f and W is omitted in the notation. We will make use of this construction with different choices of f and W.

Define the positive closed and open level sets of W by

$$\mathcal{P}_c = \{x \in \mathbb{R}^n_{\geq 0} \mid Wx = c\}, \qquad \mathcal{P}_c^+ = \{x \in \mathbb{R}^n_{>0} \mid Wx = c\}. \tag{4}$$

It follows readily that the two set are convex. By reordering the columns of W, the vector $(x_1, \dots, x_n)$ and the coordinates of f simultaneously, if necessary, we can assume without loss of generality that $\{i_1, \dots, i_d\} = \{1, \dots, d\}$. In this case, W has the block form

$$W = (I_d \quad \widehat{W}), \tag{5}$$

where $\widehat{W} \in \mathbb{R}^{d\times s}$, $s := n - d$, and I_d is the identity matrix of size d. The last s coordinates of the function φ_c come from f.

Assuming this reordering, let $\pi\colon \mathbb{R}^n \to \mathbb{R}^s$ be the projection onto the last s coordinates. Using (5), it follows that

$$Wx = c \quad \text{if and only if} \quad (x_1, \dots, x_d)^T = c - \widehat{W}(\pi(x)). \tag{6}$$

In particular, for $x, y \in \mathbb{R}^n$ fulfilling $Wx = Wy$, we have that

$$x = y \quad \text{if and only if} \quad \pi(x) = \pi(y). \tag{7}$$

If $Wf(x) = 0$, then it follows from (7) that $f(x) = 0$ if and only if $\pi(f(x)) = 0$.

Our first result concerns the Brouwer degree of φ_c. The proof of the theorem is adapted from the proof of Lemma 2 in [8] in order to account for the reduction in dimension introduced by $\mathcal{P}_c$.

Theorem 2.3. *Let $f\colon \mathbb{R}^n_{\geq 0} \to \mathbb{R}^m$ be a $\mathcal{C}^1$-function and $W \in \mathbb{R}^{d\times n}$ a matrix of rank d. Let $s := n - d$, $c \in \mathbb{R}^d$, $\mathcal{P}_c$ as in (4) and φ_c as in (3). Let B_c be an open, bounded and convex subset of $\mathbb{R}^n_{>0}$ such that*

(i) $B_c \cap \mathcal{P}_c \neq \emptyset$.

(ii) $f(x) \neq 0$ *and* $Wf(x) = 0$ *for* $x \in \mathrm{bd}(B_c) \cap \mathcal{P}_c$.

(iii) for every $x \in \mathrm{bd}(B_c) \cap \mathcal{P}_c$*, the vector* $f(x)$ *points inwards* B_c *at* x.

Then

$$\deg(\varphi_c, B_c, 0) = (-1)^s.$$

Proof. Without loss of generality, we might assume that W has the block form in (5). Choose an arbitrary point $\bar{x} \in B_c \cap \mathcal{P}_c$, which exists by assumption (i), and consider the continuous function $G\colon \mathrm{cl}(B_c) \to \mathbb{R}^n$ defined by

$$G(x) = (Wx - c, \pi(\bar{x} - x)) \in \mathbb{R}^d \times \mathbb{R}^s \cong \mathbb{R}^n,$$

where π is the projection map onto the last s coordinates of $\mathbb{R}^n$. By (5), the Jacobian of G has the block form

$$J_G(x) = \begin{pmatrix} I_d & \widehat{W} \\ 0 & -I_s \end{pmatrix}.$$

Therefore, $\det(J_G(x)) = (-1)^s$ for all x. In particular, 0 is a regular value for G. Furthermore, if $G(x) = 0$, then $x \in \mathcal{P}_c$ since $Wx = c$ and $\pi(\bar{x}) = \pi(x)$. Using (7), we conclude that $\bar{x} = x$. Since $\bar{x} \notin \mathrm{bd}(B_c)$, it follows that G does not vanish on the boundary. We apply Theorem 2.1 to compute the degree of G for 0:

$$\deg(G, B_c, 0) = \mathrm{sign}(\det(J_G(\bar{x}))) = (-1)^s.$$

Consider now the following homotopy between the functions φ_c and G:

$$\begin{array}{rcl} H\colon \mathrm{cl}(B_c) \times [0,1] & \to & \mathbb{R}^n \\ (x,t) & \mapsto & t\varphi_c(x) + (1-t)G(x). \end{array}$$

Clearly, H is continuous. To apply (A3) to find the degree of φ_c, we need to show that $H(\mathrm{bd}(B_c) \times [0,1]) \neq 0$ for all $t \in [0,1]$. Since

$$H(x,t) = (Wx - c, t\pi(f(x)) + (1-t)\pi(\bar{x} - x)),$$

$H(x,t) = 0$ implies that $Wx = c$ and hence $x \in \mathcal{P}_c$. Thus, we need to show that

$$t\pi(f(x)) + (1-t)\pi(\bar{x} - x) \neq 0 \quad \text{for all} \quad x \in \mathrm{bd}(B_c) \cap \mathcal{P}_c. \tag{8}$$

For $t = 1$, (8) follows from (7) using that $f(x) \neq 0$ and $Wf(x) = 0$ for $x \in \mathrm{bd}(B_c) \cap \mathcal{P}_c$ by assumption (ii). For $t = 0$, we have already shown that G does not vanish on the boundary of B_c.

Assume now that for $t \in (0,1)$, (8) does not hold. That is, there exists $x' \in \mathrm{bd}(B_c) \cap \mathcal{P}_c$ such that

$$\pi(f(x')) = \frac{t-1}{t}\pi(\bar{x} - x').$$

Since $x' \in \mathrm{bd}(B_c) \cap \mathcal{P}_c$, we have that $Wf(x') = 0$ and $W(\bar{x} - x') = 0$. We conclude using (7) that

$$f(x') = \frac{t-1}{t}(\bar{x} - x'). \tag{9}$$

Since $\frac{t-1}{t} < 0$, $\bar{x} \in B_c$ and $x' \in \mathrm{bd}(B_c)$, it follows from Lemma 1.1(iii) that $f(x')$ points outwards B_c at x', contradicting assumption (iii).

Therefore, $H(x,t) \neq 0$ for all $x \in \mathrm{bd}(B_c)$ and $t \in [0,1]$. As a consequence, the homotopy invariance of the Brouwer degree (A3), gives the desired result

$$\deg(\varphi_c, B_c, 0) = \deg(G, B_c, 0) = (-1)^s.$$

□

3 Chemical reaction networks

3.1 Setting

Consider a chemical reaction network with species set $\{X_1, \ldots, X_n\}$ and reactions:

$$R_j\colon \sum_{i=1}^{n} \alpha_{ij} X_i \to \sum_{i=1}^{n} \beta_{ij} X_i, \qquad j = 1, \ldots, \ell, \tag{10}$$

where α_{ij}, β_{ij} are non-negative integers. The left hand side is called the reactant complex and the right hand side the product complex.

The ODE system associated with the chemical reaction network G (as described in the main text) takes the form

$$\dot{x} = f(x) = Nv(x), \quad f\colon \mathbb{R}^n_{\geq 0} \to \mathbb{R}^n, \tag{11}$$

where $N \in \mathbb{R}^{n\times\ell}$ is the stoichiometric matrix and $v(x)$ is the vector of rate functions, which are assumed to be $\mathcal{C}^1$-functions (e.g. mass-action monomials).

We say that the network has rank s if the rank of the stoichiometric matrix is s and define $d = n - s$ to be the corank of the network. The stoichiometric compatibility classes are the convex sets $\mathcal{P}_c$ defined in (4), where W is a matrix such that the rows form a basis of $\operatorname{im}(N)^\perp$. By construction, a trajectory of (11) is confined to the stoichiometric compatibility class where its initial condition belongs to. The positive stoichiometric compatibility classes $\mathcal{P}_c^+$ are defined accordingly.

The positive solutions to the system of equations $\varphi_c(x) = 0$ with φ_c as in (3), are precisely the positive equilibria of the network in the stoichiometric compatibility class $\mathcal{P}_c$.

Let $\phi(x,t)$ denote the flow of the ODE system and let the semiflow of the ODE system be the restriction of the flow to $t \geq 0$. It is assumed that the choice of rate functions $v(x)$ is such that

$$v_j(x) = 0 \quad \text{if} \quad x_i = 0 \quad \text{for some } i \text{ with } \alpha_{ij} > 0. \tag{12}$$

In particular, mass-action kinetics fulfil this condition. Under this assumption, the non-negative and the positive orthants, $\mathbb{R}^n_{\geq 0}$ and $\mathbb{R}^n_{>0}$, are forward invariant under the ODE system (11), cf. [1, Section 16]. That is, if $x_0 \in \mathbb{R}^n_{\geq 0}$ (resp. $\mathbb{R}^n_{>0}$), then the solution to the ODE system (11) with initial condition x_0 is confined to $\mathbb{R}^n_{\geq 0}$ (resp. $\mathbb{R}^n_{>0}$):

$$x_0 \in \mathbb{R}^n_{\geq 0} \Rightarrow \phi(x_0, t) \in \mathbb{R}^n_{\geq 0}, \qquad \forall t \geq 0 \quad \text{in the interval of definition.} \tag{13}$$

Forward invariance implies that the semiflow $\phi(x,t)$ maps $\mathbb{R}^n_{\geq 0}$ to itself for any fixed $t \geq 0$ for which the solution is defined.

Since the dynamics is confined to the stoichiometric compatibility classes, this implies that for a point x' at the relative boundary of $\mathcal{P}_c$, the vector $f(x')$ points inwards $\mathcal{P}_c$. Further, both $\mathcal{P}_c$ and $\mathcal{P}_c^+$ are also forward invariant sets. Recall that these are convex sets.

3.2 Conservative and dissipative networks

Definition 3.1. A chemical reaction network is conservative if $\operatorname{im}(N)^\perp$ contains a positive vector, that is, if $\mathbb{R}^n_{>0} \cap \operatorname{im}(N)^\perp \neq \emptyset$.

A network is *conservative* if and only if the stoichiometric compatibility classes $\mathcal{P}_c$ are compact subsets of $\mathbb{R}^n_{\geq 0}$ [2].

Definition 3.2. Consider a network with associated ODE system $\dot{x} = Nv(x)$. The semiflow of the network is *dissipative* if for all $c \in \mathbb{R}^d$ such that $\mathcal{P}_c^+ \neq \emptyset$, there exists a compact set $K_c \subseteq \mathcal{P}_c$ such that $\phi(x,t) \in K_c$ for all $x \in \mathcal{P}_c$ and $t \geq t(x)$, for some $t(x) \geq 0$. That is, all trajectories in $\mathcal{P}_c$ enter K_c at some point.

The set K_c is called *attracting* (and sometimes *absorbing*) [6]. Equivalently, the semiflow of a network is dissipative if all trajectories are *eventually uniformly bounded*, that is, there exists a constant $k > 0$ such that

$$\limsup_{t\to+\infty} x_i(t) \leq k$$

for all $i = 1, \ldots, n$ and all initial conditions in $\mathcal{P}_c$, provided that $\mathcal{P}_c^+ \neq \emptyset$ for c arbitrary.

If the semiflow of the network is dissipative, then the unique solution to the ODE system (11) for a given initial condition is defined for all $t \geq 0$, in which case the semiflow is said to be forward complete.

Lemma 3.3. *Consider a network with a dissipative semiflow and let $c \in \mathbb{R}^d$ such that $\mathcal{P}_c^+ \neq \emptyset$. Then the following holds:*

(i) An attracting set K_c can be chosen such that $K_c \cap \mathbb{R}^n_{>0}$ is non-empty, that is, $K_c \not\subseteq \mathrm{bd}(\mathbb{R}^n_{\geq 0})$.

(ii) All ω-limit points in $\mathcal{P}_c$ of the system are contained in K_c. In particular, all positive equilibria in $\mathcal{P}_c$ belong to K_c.

(iii) There exists an attracting set K'_c such that $K_c \subseteq K'_c$, K'_c is forward invariant and all ω-limit points outside the boundary of $\mathbb{R}^n_{\geq 0}$ are in the interior of K'_c (relatively to $\mathcal{P}_c$).

Proof. (i) Consider an attracting set $K''_c \subseteq \mathcal{P}_c$ and assume that $K''_c \subseteq \mathrm{bd}(\mathbb{R}^n_{\geq 0})$. Since $\mathcal{P}_c^+ \neq \emptyset$, there exists a compact set $K_c \subseteq \mathcal{P}_c$ that includes K''_c and such that $K_c \cap \mathbb{R}^n_{>0}$ is non-empty. This set is also an attracting set.

(ii) If it were not the case, there would exist an ω-limit point $x' \in \mathcal{P}_c \backslash K_c$, a trajectory $\phi(x,t)$ and a sequence of time points t_i such that $\lim_{i\to\infty} t_i = \infty$ and $\lim_{i\to\infty} \phi(x,t_i) = x'$. As K_c is closed, there exists an open ball $B_\epsilon(x')$ in $\mathbb{R}^n$ such that $B_\epsilon(x') \cap K_c = \emptyset$ and $\phi(x,t) \in B_\epsilon(x')$ for arbitrary many time points. However, this contradicts that K_c is an attracting set.

(iii) By (ii) and choosing K_c potentially larger, all ω-limit points outside the boundary of $\mathbb{R}^n_{\geq 0}$ are in the interior of K_c (relatively to $\mathcal{P}_c$). The existence of an attracting set K'_c that includes K_c and is forward-invariant is proven in the first part of the proof of Lemma 2 in [6]. In the notation of [6], $K'_c = K^+$. □

The semiflow of a conservative network is dissipative. Indeed, it is sufficient to take $K_c = \mathcal{P}_c$, since $\mathcal{P}_c$ is compact. If the network is not conservative, then the semiflow associated with the network might still be dissipative (see Example "Gene transcription network" in the main text). However, in general, it is not straightforward to show that. In some cases it is possible to prove dissipativity by constructing a suitable Lyapunov function. It is the idea underlying the proof of the next proposition.

Proposition 3.4. *Assume that for all $c \in \mathbb{R}^d$ such that $\mathcal{P}_c^+ \neq \emptyset$, there exists a vector $\omega \in \mathbb{R}^n_{>0}$ and a real number $r > 0$ such that $\omega \cdot f(x) < 0$ for all $x \in \mathcal{P}_c$ with $||x|| \geq r$, where $||\cdot||$ is any norm. (Note that ω and r might depend on c.) Then the semiflow of the network is dissipative.*

Proof. Let $c \in \mathbb{R}^d$ with $\mathcal{P}_c^+ \neq \emptyset$ and let ω be as given in the statement. Define

$$V(x) = \sum_{i=1}^{n} \omega_i x_i \quad \text{for} \quad x \in \mathbb{R}^n_{\geq 0}.$$

The function $V(x)$ satisfies $V(0) = 0$ and $V(x) > 0$ for all $x \in \mathbb{R}^n_{\geq 0}$, different from 0. Further, for $||x|| \geq r$ and $x \in \mathcal{P}_c$, $\dot{V}(x) = \nabla V \cdot f(x) = \omega \cdot f(x) < 0$ by assumption. Thus, $V(x)$ is a strict Lyapunov function and $V(\phi(x,t))$ is strictly decreasing along trajectories $\phi(x,t)$ in $\mathcal{P}_c$ as long as $||\phi(x,t)|| \geq r$. Choose $R > 0$ such that

$$\{x \in \mathbb{R}^n_{\geq 0} \mid ||x|| \leq r\} \subseteq \{x \in \mathbb{R}^n_{\geq 0} \mid V(x) \leq R\},$$

and define $K_c = \{x \in \mathbb{R}^n_{\geq 0} \mid V(x) \leq R\} \cap \mathcal{P}_c$. The set K_c is compact by construction and forward invariant since $\dot{V}(x) < 0$ for all $||x|| \geq r$. Further, all trajectories eventually enter K_c within finite time, that is, K_c is attracting. Indeed, if this were not the case, then there would exist $x \in \mathcal{P}_c$, $x \notin K_c$ (hence $||x|| > r$) such that $V(\phi(x,t))$ is decreasing for all $t \geq 0$ in the interval of definition and bounded below by R. As a consequence, the trajectory is defined for all $t \geq 0$ and $(*)$ $\lim_{t\to\infty} V(\phi(x,t)) = R' \geq R$ for some R'. Hence $\phi(x,t)$ is in $B_\epsilon := \{x \mid V(x) \leq R' + \epsilon\}$ for large t (and any $\epsilon > 0$). Since B_ϵ is compact it follows that the semiflow $\phi(x,t)$ has at least one ω-limit point in B_ϵ. By virtue of $(*)$, all ω-limit points x' of $\phi(x,t)$ must fulfil $V(x') = R'$. Further, the set of ω-limit points is forward invariant and since $V(x') = R'$ it must be that $\dot{V}'(x') = 0$. This contradicts the assumption that $\dot{V}'(x) < 0$ for all x with $||x|| \geq r$. We conclude that there exists $t(x) \geq 0$ such that $\phi(x,t) \in K_c$ for all $x \in \mathcal{P}_c$ and $t \geq t(x)$. Hence, the semiflow is dissipative. □

3.3 Degree for dissipative semiflows

The main results to establish a characterization of regions of multistationarity (Theorem 4.1) are Theorem 2.1 and the theorem below. The proof of the theorem relies on Theorem 2.3 and ideas developed in [6].

Theorem 3.5. *Consider a network of rank s with an associated ODE system $\dot{x} = f(x)$ where $f(x) = Nv(x)$ as in (11). Assume (12) holds on the rate functions and let $W \in \mathbb{R}^{d\times n}$, $d = n - s$, be a row reduced matrix such that the rows of W form a basis of $\operatorname{im}(N)^\perp$. Let $c \in \mathbb{R}^d$ such that $\mathcal{P}_c^+ \neq \emptyset$. Further, assume that:*

- *The semiflow of the network is dissipative, and that*
- *$f(x) \neq 0$ for all $x \in \operatorname{bd}(\mathbb{R}^n_{\geq 0}) \cap \mathcal{P}_c$. That is, there are no boundary equilibria in $\mathcal{P}_c$.*

Then there exists an open bounded and convex set $B_c \subseteq \mathbb{R}^n_{>0}$ that contains all positive equilibria of the network in the stoichiometric compatibility class $\mathcal{P}_c$, and such that

$$\deg(\varphi_c, B_c, 0) = (-1)^s,$$

where φ_c is defined in (3) from f and W.

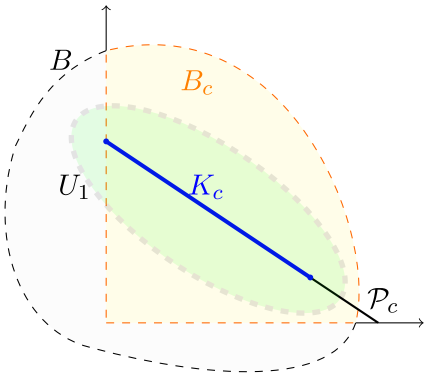

Figure 1: Step (A). The set $\mathcal{P}_c$ is the straight line connecting the two axis. The compact attracting set K_c is depicted in blue. The set $B \subseteq \mathbb{R}^n$ is an open set containing K_c and $B_c = B \cap \mathbb{R}^n_{>0}$ is the restriction of B to the positive orthant (shown in orange), such that B_c is open. Hence K_c is contained in B_c, except for points on the boundary $K_c \cap \mathrm{bd}(\mathbb{R}^n_{\geq 0})$, hence also $B_c \cap \mathcal{P}_c \neq \emptyset$. Step (B). The open set $U_1 \subseteq \mathbb{R}^n$ (in green) is chosen such that $K_c \subseteq U_1 \subseteq B$. In the $\mathcal{C}^1$-partition of unit, the support of ψ_1 is in U_1 and that of ψ_2 is in $\mathbb{R}^n \setminus K_c$.

Proof. The idea of the proof is to construct a function g defined on $\mathbb{R}^n_{\geq 0}$ and a set $B_c \subseteq \mathbb{R}^n_{>0}$ such that the conditions of Theorem 2.3 are fulfilled for g, W and B_c. If we let φ_c^g be the function φ_c in (3) constructed from the function g and W, this will imply that $\deg(\varphi_c^g, B_c, 0) = (-1)^s$. Subsequently, we will use homotopy invariance to conclude that also $\deg(\varphi_c, B_c, 0) = (-1)^s$.

The function g will be defined as

$$g(x) = \frac{1}{T}(\phi(x,T) - x) + T\rho(x),$$

where $\phi(x,t)$ is the semiflow of $\dot{x} = f(x)$, K_c is a suitably chosen attracting set, T is the maximum entrance time into K_c from a specific set, and $\rho(x)$ is an auxiliary function with certain useful properties (see below).

The proof is divided into four steps. In step (A) we define the set B_c, choose K_c and find basic properties of B_c and K_c. In step (B), we construct the function ρ. In step (C), we properly define g and show that g, B_c and W have the required properties to apply Theorem 2.3. In step (D) we show that φ_c^g and φ_c are homotopy equivalent and conclude the proof of the theorem using the homotopy invariance of the Brouwer degree.

(A) Let $K_c \subseteq \mathcal{P}_c$ be as in Definition 3.2, that is, a compact attracting set of all trajectories with initial condition in $\mathcal{P}_c$. According to Lemma 3.3, K_c can be chosen such that K_c is forward invariant, $K_c \cap \mathbb{R}^n_{>0} \neq \emptyset$, and all ω-limit points in $\mathcal{P}_c^+$ are interior points of K_c (relatively to $\mathcal{P}_c$).

Let $B \subseteq \mathbb{R}^n$ be an open, bounded and convex set containing K_c, that is, $K_c \subseteq B$. Let $B_c = \mathbb{R}^n_{>0} \cap B$. Then B_c is also open, bounded and convex. Since $K_c \subseteq \mathbb{R}^n_{\geq 0} \cap B$, then B_c contains all points in K_c except those on the boundary $K_c \cap \mathrm{bd}(\mathbb{R}^n_{\geq 0})$. Further,

$$K_c \subseteq \mathrm{cl}(B_c) \subseteq \mathbb{R}^n_{\geq 0}, \quad \text{and} \quad K_c \cap \mathrm{bd}(B_c) = K_c \cap \mathrm{bd}(\mathbb{R}^n_{\geq 0}),$$

see Figure 1. Since $\emptyset \neq K_c \cap \mathbb{R}^n_{>0} = B \cap K_c \cap \mathbb{R}^n_{>0} = B_c \cap K_c \subseteq B_c \cap \mathcal{P}_c$, then

$$B_c \cap \mathcal{P}_c \neq \emptyset.$$

Since $f(x) \neq 0$ for all $x \in \mathrm{bd}(\mathbb{R}^n_{\geq 0}) \cap \mathcal{P}_c$ by assumption and K_c contains all zeros of f in $\mathcal{P}_c$, then B_c contains all zeros of f in $\mathcal{P}_c$, that is

$$\{x \in \mathcal{P}_c \mid f(x) = 0\} \subseteq B_c. \tag{14}$$

(B) The function $\rho\colon \mathbb{R}^n_{\geq 0} \to \mathbb{R}^n$ in the definition of g is defined such that it has the following properties:

(i) $\rho(x)$ points inwards B_c for all $x \in \mathrm{bd}(B_c) \cap \mathcal{P}_c$.

(ii) $\rho(x) = 0$ for $x \in \mathbb{R}^n_{>0} \cap \mathrm{bd}(B_c) \cap \mathcal{P}_c$.

(iii) $\rho(x) \neq 0$ for $x \in K_c \cap \mathrm{bd}(B_c)$.

(iv) $W\rho(x) = 0$ for all $x \in \mathrm{bd}(B_c) \cap \mathcal{P}_c$.

We first construct two other functions $\widetilde{\rho}$ and ψ_1, and subsequently define $\rho\colon \mathbb{R}^n_{\geq 0} \to \mathbb{R}^n$ as the product $\rho = \widetilde{\rho}\psi_1$. Let $\widetilde{x} \in K_c \cap \mathbb{R}^n_{>0}$ and define $\widetilde{\rho}\colon \mathbb{R}^n \to \mathbb{R}^n$ as $\widetilde{\rho}(x) := \widetilde{x} - x$. Let $U_1 \subsetneq B$ be an open set containing K_c (which exists since B is open), see Figure 1. Consider the open cover of $\mathbb{R}^n$ given by U_1 and $U_2 = \mathbb{R}^n \setminus K_c$, such that $U_1 \cap U_2 \neq \emptyset$ and $U_1 \cup U_2 = \mathbb{R}^n$. Choose a $\mathcal{C}^1$-partition of unit $\psi_1, \psi_2\colon \mathbb{R}^n \to [0,1]$ associated with this open cover. This implies in particular that the support of ψ_i is included in U_i and $\psi_1(x) + \psi_2(x) = 1$ for all x.

Define $\rho\colon \mathbb{R}^n_{\geq 0} \to \mathbb{R}^n$ by $\rho(x) = \psi_1(x)\widetilde{\rho}(x)$, $x \in \mathbb{R}^n_{\geq 0}$ (note the restriction to $\mathbb{R}^n_{\geq 0}$). This function fulfils properties (i)-(iv) above. Property (i): Follows by definition of $\rho(x) = \psi_1(x)(\widetilde{x} - x)$, $\psi_1(x) \geq 0$ and Lemma 1.1(iii), using that $\widetilde{x} \in B_c$ and $x \in \mathrm{bd}(B_c)$. Property (ii): Since the support of ψ_1 is contained in U_1, $\psi_1(x) = 0$ for all $x \notin U_1$, in particular for all $x \in \mathbb{R}^n_{>0} \cap \mathrm{bd}(B_c) \cap \mathcal{P}_c$, since $\mathbb{R}^n_{>0} \cap \mathrm{bd}(B_c) \subseteq \mathrm{bd}(B)$ and $\mathrm{bd}(B) \cap U_1 = \emptyset$. Property (iii): Similarly, $\psi_1(x) = 1$ (since $\psi_2(x) = 0$) for all $x \notin U_2 = \mathbb{R}^n \setminus K_c$, that is, for all $x \in K_c$; hence $\rho(x) \neq 0$ for $x \in K_c \cap \mathrm{bd}(B_c)$ since $\widetilde{x} \notin \mathrm{bd}(B_c)$. Property (iv): $W\rho(x) = \psi_1(x)W(\widetilde{x} - x) = 0$ as $x, \widetilde{x} \in \mathcal{P}_c$.

(C) Let T be defined as the maximum of the entry times to K_c from any $x \in \mathrm{cl}(B_c) \cap \mathcal{P}_c$. The time T is finite because $\mathrm{cl}(B_c) \cap \mathcal{P}_c$ is compact and the semiflow is dissipative with respect to K_c. Note that once a trajectory is in K_c, it stays there since K_c is forward invariant Redefine T to be any positive number if $T = 0$.

We define

$$g\colon \mathbb{R}^n_{\geq 0} \to \mathbb{R}^n, \quad g(x) := \frac{1}{T}(\phi(x,T) - x) + T\rho(x),$$

Observe that $Wg(x) = 0$ for all $x \in \mathrm{bd}(B_c) \cap \mathcal{P}_c$, using property (iv) in step (B) and that $\phi(x,T), x \in \mathcal{P}_c$. By definition of T, $\phi(x,T) \in \mathrm{cl}(B_c) \cap \mathcal{P}_c$ if $x \in \mathrm{cl}(B_c) \cap \mathcal{P}_c$ and hence $\frac{1}{T}(\phi(x,T) - x)$ points inwards B_c at $x \in \mathrm{bd}(B_c) \cap \mathcal{P}_c$ by convexity of $\mathrm{cl}(B_c)$. Also $T\rho(x)$ points inwards B_c at x by property (i) in step (B). Hence, $g(x)$ points inwards B_c at $x \in \mathrm{bd}(B_c) \cap \mathcal{P}_c$ by convexity again.

Therefore, the function g, together with B_c and W, fulfil the conditions of Theorem 2.3. By letting φ_c^g be the function φ_c in (3) constructed from g and W, we conclude that

$$\deg(\varphi_c^g, B_c, 0) = (-1)^s.$$

(D) We define a homotopy between φ_c and φ_c^g on $\mathrm{cl}(B_c) \times [0,T]$ by

$$H(x,t) = \begin{cases} \varphi_c(x) & \text{if} \quad t = 0 \\ \left(Wx - c, \frac{1}{t}\pi(\phi(x,t) - x) + t\pi(\rho(x))\right) & \text{if} \quad 0 < t \leq T. \end{cases}$$

The function $H(x,t)$ is continuous since $\phi(x,t)$ is differentiable and is the semiflow of $\dot{x} = f(x)$. Note that $H(x,0) = \varphi_c(x)$ and $H(x,T) = (Wx - c, \pi(g(x))) = \varphi_c^g(x)$. Thus $H(x,t)$ is a homotopy between $\varphi_c(x)$ and $\varphi_c^g(x)$. We need to show that $H(x,t)$ does not vanish on the boundary $\mathrm{bd}(B_c)$.

If $H(x,0) = \varphi_c(x) = 0$, then $x \in \mathcal{P}_c$ is an equilibrium of the ODE system. Hence $H(x,0)$ does not vanish on $\mathrm{bd}(B_c)$ since B_c contains all zeros of f in $\mathcal{P}_c$, see (14). Now let $x' \in \mathrm{bd}(B_c)$ and assume that $H(x',t) = 0$ for some $t \in (0,T]$. It follows that $x' \in \mathcal{P}_c$, hence

$$x' \in \mathrm{bd}(B_c) \cap \mathcal{P}_c, \quad \text{and} \quad \pi(\phi(x',t) - x') = -t^2\pi(\rho(x')). \tag{15}$$

Using (7) and property (iv) in step (B) we have that

$$\phi(x',t) = x' - t^2\rho(x'). \tag{16}$$

By construction of K_c, all fixed points and periodic orbits are contained in K_c. If $\rho(x') = 0$, then (16) implies $x' \in K_c \cap \mathrm{bd}(B_c)$ as $x' \in \mathrm{bd}(B_c)$ by assumption. However, this contradicts property (iii) in step (B). Hence, it must be the case that $\rho(x') \neq 0$.

Using that $x' \in \mathrm{bd}(B_c) \cap \mathcal{P}_c$ from (15) and $\rho(x') \neq 0$, we conclude that $x' \in \mathrm{bd}(\mathbb{R}^n_{\geq 0})$ by property (ii) in step (B), since $x' \notin \mathbb{R}^n_{>0} \cap \mathrm{bd}(B_c) \cap \mathcal{P}_c$. It follows that there exists i such that $x'_i = 0$ and we have

$$\phi(x',t)_i = x'_i - t^2\rho(x')_i = x'_i - t^2\psi_1(x')\widetilde{\rho}(x')_i = -t^2\psi_1(x')\widetilde{x}_i < 0.$$

Here we have used that $\psi_1(x') \neq 0$, since $\rho(x') = \psi_1(x')\widetilde{\rho}(x') \neq 0$ and $\psi_1(x')$ is a scalar. Now, by the inequality above, $\phi(x',t)$ does not belong to $\mathbb{R}^n_{\geq 0}$. However, this contradicts the forward invariance of $\mathbb{R}^n_{\geq 0}$ with respect to the flow. Therefore, H does not vanish on $\mathrm{bd}(B_c) \times [0,T]$.

With this in place, homotopy invariance of the Brouwer degree implies that

$$\deg(\varphi_c, B_c, 0) = \deg(H(x,0), B_c, 0) = \deg(H(x,T), B_c, 0) = \deg(\varphi_c^g, B_c, 0) = (-1)^s.$$

□

Remark 3.6. The statement and proof of the theorem focus exclusively on one stoichiometric compatibility class, that is, on a fixed value $c \in \mathbb{R}^d$. Therefore, if a semiflow admits an attracting set in one specific stoichiometric compatibility class (and not necessarily in all), then the theorem and computation of the Brouwer degree holds for this specific class.

4 Multistationarity in dissipative networks

In this section we prove the theorem and corollaries stated in the main text, which are consequences of Theorem 3.5 from the previous section.

Consider the Jacobian of the map $\varphi_c(x)$. Because $\varphi_c(x)$ is independent of c, we denote the Jacobian by $M(x)$. The i-th row of this matrix is given as

$$M(x)_i := J_{\varphi_c}(x)_i = \begin{cases} J_{f_i}(x) & i \notin \{i_1,\dots,i_d\} \\ W_i & i \in \{i_1,\dots,i_d\}, \end{cases}$$

where W_i is the i-th row of W. That is, one can think of $M(x)$ as being the matrix obtained from the Jacobian of $f(x)$, with the i_j-th row, $j = 1,\dots,d$, replaced by the j-th row of W.

An equilibrium x^* is said to be *non-degenerate* if $M(x^*)$ has rank n, that is, if $\det(M(x^*)) \neq 0$.

Theorem 4.1. *Assume the reaction rate functions fulfil* (12)*, let* $s = rank(N)$ *and let* $\mathcal{P}_c$ *be a stoichiometric compatibility class such that* $\mathcal{P}_c^+ \neq \emptyset$ *(where* $c \in \mathbb{R}^d$*). Further, assume that*
(i) The semiflow of the network is dissipative.
(ii) There are no boundary equilibria in $\mathcal{P}_c$*.*
Then the following holds:
(A') **Uniqueness of equilibria.** *If*

$$\operatorname{sign}(\det(M(x))) = (-1)^s \quad \textit{for all equilibria} \quad x \in V \cap \mathcal{P}_c^+,$$

then there is exactly one positive equilibrium in $\mathcal{P}_c$*. Further, this equilibrium is non-degenerate.*
(B') **Multiple equilibria.** *If*

$$\operatorname{sign}(\det(M(x))) = (-1)^{s+1} \quad \textit{for some equilibrium} \quad x \in V \cap \mathcal{P}_c^+,$$

then there are at least two positive equilibria in $\mathcal{P}_c$*, at least one of which is non-degenerate. If all positive equilibria in* $\mathcal{P}_c$ *are non-degenerate, then there are at least three and always an odd number.*

Proof. The hypotheses ensure that we can apply Theorem 3.5. Therefore choose an open bounded convex set $B_c \subset \mathbb{R}^n_{>0}$ that contains all positive equilibria of the network in the stoichiometric compatibility class $\mathcal{P}_c$ and such that

$$\deg(\varphi_c, B_c, 0) = (-1)^s.$$

Let V_c be the set of positive equilibria in the stoichiometric compatibility class $\mathcal{P}_c$. Note that

$$V_c = \{x \in B_c \mid \varphi_c(x) = 0\}.$$

(A') Since $\operatorname{sign}(\det(M(x))) = (-1)^s \neq 0$ for all equilibria in $\mathcal{P}_c$, 0 is a regular value for φ_c. We can therefore apply Theorem 2.1 and obtain

$$(-1)^s = \sum_{x \in V_c} \operatorname{sign}(\det(M(x))) = (-1)^s(\#V_c),$$

where $\#V_c$ is the cardinality of V_c. We conclude that $\#V_c = 1$ and therefore that there exists a unique positive equilibrium in the stoichiometric compatibility class. Furthermore, since $\operatorname{sign}(\det(M(x))) \neq 0$ for all equilibria, the equilibrium is non-degenerate.

(B') Let $x^* \in V_c$ be such that $\operatorname{sign}(\det(M(x^*))) = (-1)^{s+1}$. If 0 is a regular value for $\varphi_c(\cdot)$, then the equality

$$(-1)^s = \sum_{x \in V_c} \operatorname{sign}(\det(M(x))) = (-1)^{s+1} + \sum_{x \in V_c,\ x \neq x^*} \operatorname{sign}(\det(M(x)))$$

implies that there must exist at least two other points $x', x'' \in V_c$, such that

$$\operatorname{sign}(\det(M(x'))) = \operatorname{sign}(\det(M(x'')) = (-1)^s,$$

that is, there are at least three positive equilibria in $\mathcal{P}_c$, all of which are non-degenerate. In this case by Corollary 2.2, there is an odd number of equilibria and they are all non-degenerate.

Assume now that 0 is not a regular value for φ_c. Then there must exist another positive equilibrium x' in $\mathcal{P}_c$ for which the Jacobian of $\varphi_c(x')$ is singular. This implies that there are at least two positive equilibria in $\mathcal{P}_c$, x^* and x', one of which is non-degenerate. □

In typical applications we find an odd number of equilibria (≥ 3), all of which are non-degenerate. Observe that the hypothesis for Part (A) holds if the sign of $\det(M(x))$ is $(-1)^s$ for all x in a set containing the positive equilibria. In particular, this is the case if $\det(M(x)) = (-1)^s$ for all $x \in \mathbb{R}^n_{>0}$.

We assume now that the positive solutions to the system $f(x) = 0$ (with $f(x)$ as in (11)) admit a parameterization

$$\begin{aligned} \Phi\colon \mathbb{R}^m_{>0} &\to \mathbb{R}^n_{>0} \\ \hat{x} = (\hat{x}_1, \dots, \hat{x}_m) &\mapsto (\Phi_1(\hat{x}), \dots, \Phi_n(\hat{x})), \end{aligned} \tag{17}$$

for some $m < n$. That is, we assume that we can express $x_1, \dots, x_n$ at equilibrium as functions of $\hat{x}_1, \dots, \hat{x}_m$:

$$x_i = \Phi_i(\hat{x}_1, \dots, \hat{x}_m), \qquad i = 1, \dots, n,$$

such that $x_1, \dots, x_n$ are positive if $\hat{x}_1, \dots, \hat{x}_m$ are positive.

For mass-action kinetics, the equation $f(x) = 0$ results in $s = n - d$ polynomial equations in n unknowns, which generically would lead to a d-dimensional parameterization and $m = d$ (if such a parameterization exists).

When such a parameterization exists, then positive values of $\hat{x}_1, \dots, \hat{x}_m$ determine uniquely a positive equilibrium. This equilibrium then belongs to the stoichiometric compatibility class given by

$$c := W\Phi(\hat{x}).$$

Reciprocally, given c, the positive solutions to $\varphi_c(x) = 0$ are in one-to-one correspondence with the positive solutions to the equation $c = W\Phi(\hat{x})$.

As before, we let $W \in \mathbb{R}^{d\times n}$ be a row-reduced matrix whose rows form a basis of $\mathrm{im}(N)^\perp$. Let $i_1, \dots, i_d$ be the indices of the first non-zero coordinate of each row. Let $\pi\colon \mathbb{R}^n \to \mathbb{R}^s$ denote the projection onto the coordinates with indices different from $i_1, \dots, i_d$. We do not reorder the coordinates now to ensure that $\{i_1, \dots, i_d\} = \{1, \dots, d\}$, because we have already chosen a convenient order of the free variables of the parameterization.

We next consider the determinant of $M(x)$ and use the parameterization (17) to substitute the values of $x_1, \dots, x_n$ by their expressions as functions of $\hat{x}_1, \dots, \hat{x}_m$. We define

$$a(\widehat{x}) = \det(M(\Phi(\widehat{x}))). \tag{18}$$

Corollary 4.2. *Assume the reaction rate functions fulfil* (12) *and let $s = \mathrm{rank}(N)$. Further, assume that*

(i) The semiflow of the network is dissipative.
(ii) The set of positive equilibria admits a positive parameterization as in (17).
(iii) There are no boundary equilibria in $\mathcal{P}_c$, for all $c \in \mathbb{R}^d$ such that $\mathcal{P}_c^+ \neq \emptyset$.

Then the following holds.

(A) **Uniqueness of equilibria.** *If* $\mathrm{sign}(a(\hat{x})) = (-1)^s$ *for all $\hat{x} \in \mathbb{R}^m_{>0}$, then there is exactly one positive equilibrium in each $\mathcal{P}_c$ with $\mathcal{P}_c^+ \neq \emptyset$. Further, this equilibrium is non-degenerate.*

(B) **Multiple equilibria.** *If* $\mathrm{sign}(a(\hat{x})) = (-1)^{s+1}$ *for some $\hat{x} \in \mathbb{R}^m_{>0}$, then there are at least two positive equilibria in $\mathcal{P}_c$, at least one of which is non-degenerate, where $c := W\Phi(\hat{x})$. If all positive equilibria in $\mathcal{P}_c$ are non-degenerate, then there are at least three equilibria and always an odd number.*

Proof. Given c, note that Φ induces a bijection between the sets

$$V_c \quad \text{and} \quad \mathcal{S}_c := \{\widehat{x} \in \mathbb{R}^m_{>0} \mid c = W\Phi(\widehat{x})\}.$$

An element of $\mathcal{S}_c$ corresponds to a positive equilibrium in the stoichiometric compatibility class $\mathcal{P}_c$.

(A) Consider a stoichiometric compatibility class $\mathcal{P}_c$ defined by c such that $\mathcal{P}_c^+ \neq \emptyset$. Let $x \in V_c$ and $\hat{x}$ such that $x = \Phi(\hat{x})$. Then

$$\det(M(x)) = \det(M(\Phi(\hat{x}))) = a(\widehat{x}).$$

By hypothesis $\text{sign}(\det(M(x))) = \text{sign}(a(\widehat{x})) = (-1)^s$. Since this holds for all equilibria in V_c, Theorem 4.1(A') gives that there is exactly one positive equilibrium in $\mathcal{P}_c$, which is non-degenerate.

(B) Let $\widehat{x}$ be such that $\text{sign}(a(\widehat{x})) = (-1)^{s+1}$ and let c be defined as in the statement of the theorem. Then $x = \Phi(\widehat{x})$ is a positive equilibrium in V_c for which the sign of $\det(M(x))$ is $(-1)^{s+1}$. Theorem 4.1(B') gives the desired conclusion. □

5 Details on the steps of the procedure

In this section we expand further on how to check step 3 and 7 of the algorithm.

5.1 On siphons and boundary equilibria

A proof of Proposition 2 in the main text for mass-action kinetics can be found in [11], where strategies to find siphons are also detailed. The proof in [11] is however valid for general kinetics fulfilling assumption (12) (see [9, Prop. 2]). Different algorithms developed in Petri Net theory can be applied to find the siphons of a reaction network.

For large networks, the task of finding the siphons can be daunting. A way to reduce the complexity of the computation is by the removal of intermediate species and catalysts [9]. We explain the key aspects of this reduction method here. The method is used in the examples below.

The first reduction concerns **removal of intermediates.** Intermediates are species in the network that do not appear interacting with any other species, are produced in at least one reaction, and consumed in at least one reaction. For example the species ES_0 in the reaction network

$$S_0 + E \rightleftharpoons ES_0 \rightleftharpoons S_1 + E \tag{19}$$

is an intermediate.

Given a network, we obtain a reduced network by "removing" some intermediates, one at a time. This is done in the following way. Say we want to remove an intermediate Y from the network. We remove all reactions of the original network that involve Y and add a reaction

$$y \to y' \qquad \text{whenever} \qquad y \to Y \to y' \quad \text{with} \quad y \neq y'$$

belongs to the original network. Here y and y' are the reactant complex of a reaction $y \to Y$ and product complex of a reaction $Y \to y'$, respectively.

To illustrate this, we consider the removal of the intermediate ES_0 in the network (19). The reactions of the reduced network are obtained by considering all length 2 paths of the original network that go through ES_0. We have two such paths:

$$S_0 + E \longrightarrow ES_0 \longrightarrow S_1 + E \qquad \text{and} \qquad S_1 + E \longrightarrow ES_0 \longrightarrow S_0 + E.$$

By "collapsing" these paths we obtain the reactions

$$S_0 + E \longrightarrow S_1 + E \qquad \text{and} \qquad S_1 + E \longrightarrow S_0 + E. \tag{20}$$

Clearly the process could be repeated now by choosing other intermediates of the network (if any). In this way we can obtain reduced networks by removing several intermediates.

The second reduction concerns **removal of catalysts.** Catalysts are species that whenever they appear in a reaction, then they appear at both sides and with the same stoichiometric coefficient. For example, E in the reaction network (20) is a catalyst. Catalysts are actually defined in more generality in [9], but we restrict to this scenario to keep the discussion simple. Catalysts are removed from a network by literally removing them from the reactions where they appear. Removal of E in the reaction network (20) yields the reaction network

$$S_0 \rightleftharpoons S_1. \tag{21}$$

This network has one minimal siphon, namely $\{S_0, S_1\}$, and $s_0 + s_1 = c$ is a conservation relation. By Proposition 2 in the main text it does not admit boundary equilibria in stoichiometric compatibility classes with non-empty positive part. The next proposition allows us to conclude that the original network in (19) neither admits boundary equilibria in stoichiometric compatibility classes with non-empty positive part.

Proposition 5.1 (Theorems 1 and 2 in [9]). *Let G be a network and G' be a network obtained after iterative removal of intermediates or catalysts from G as described above. Each minimal siphon of G contains the support of a positive conservation relation if and only if this is the case for G'.*

In several cases, removal of intermediates and catalysts yields a so-called *monomolecular network*. That is, a network whose complexes agree with some species or the complex zero. For example, the network in (21) is monomolecular. In this case, checking the hypothesis of Proposition 2 in the main text is straightforward, in view of the next lemma.

Lemma 5.2 (Proposition 3 in [9]). *Let G be a monomolecular network. Each minimal siphon of G contains the support of a positive conservation relation if and only if all connected components of G are strongly connected.*

The network in (21) is clearly strongly connected. Thus, we do not need to find the siphons of the network to conclude that each of its minimal siphons contains the support of a positive conservation relation and thereby conclude that (19) does not admit boundary equilibria in stoichiometric compatibility classes with non-empty positive part.

Corollary 5.3. *Let G be a network and G' be a network obtained after iterative removal of intermediates or catalysts from G as described above. If G' is a monomolecular network with all connected components strongly connected, then G has no boundary equilibria in any stoichiometric compatibility class $\mathcal{P}_c$ such that $\mathcal{P}_c^+ \neq \emptyset$.*

For example, consider the "gene transcription network" of the main text. The species X_5 and X_7 are intermediates. The reaction network obtained upon their removal is:

$$X_1 \longrightarrow X_1 + X_3 \qquad X_3 \longrightarrow 0 \qquad X_2 \longrightarrow X_2 + X_4 \qquad X_4 \longrightarrow 0 \qquad 2X_4 \rightleftharpoons X_6.$$

For this network, X_6 is an intermediate and X_1, X_2 are catalysts. Removal of these three species yields the reaction network

$$X_3 \rightleftharpoons 0 \rightleftharpoons X_4.$$

This is a strongly connected monomolecular network. By Corollary 5.3, there are no boundary equilibria in any $\mathcal{P}_c$ as long as $\mathcal{P}_c^+ \neq \emptyset$. We have reached the same conclusion as in the main text without the need of finding the minimal siphons of the network.

5.2 Newton polytope

We write a multivariate polynomial $f(x) \in \mathbb{R}[x_1, \dots, x_n]$ as a sum of monomials:

$$f(x) = \sum_{\alpha \in \mathbb{N}^n} c_\alpha x^\alpha,$$

where $x^\alpha = x_1^{\alpha_1} \dots x_n^{\alpha_n}$ and $c_\alpha \in \mathbb{R}$, for which only a finite number are non-zero.

The *Newton polytope of* $f(x)$, denoted by $\mathcal{N}(f)$, is a closed convex set in $\mathbb{R}^n$, defined as the convex hull of the exponents $\alpha \in \mathbb{N}^n$ for which $c_\alpha \neq 0$ (See [10, Section 2] for a definition of convex hull). The set of vertices of $\mathcal{N}(f)$ is a subset of the set of points α for which $c_\alpha \neq 0$.

The following is a well-known fact about the Newton polytope of a polynomial. The proof of the fact is constructive and provides an explicit way to find $\widehat{x}$ in Corollary 4.2(B). Thus it offers a way to find stoichiometric compatibility classes (i.e. values of c) for which multistationarity exists.

Proposition 5.4. *Let* $f(x) = \sum_{\alpha \in \mathbb{N}^n} c_\alpha x^\alpha$ *and let* α' *be a vertex of* $\mathcal{N}(f)$. *Then there exists* $x' \in \mathbb{R}^n_{>0}$ *such that*

$$\operatorname{sign}(f(x')) = \operatorname{sign}(c_{\alpha'}).$$

Proof. By hypothesis $c_{\alpha'} \neq 0$. Since α' is a vertex in a bounded convex polytope, there exists a separating hyperplane $\omega \cdot x = T$ that intersects the polytope only in α' and such that $\omega \cdot x' < T$ for any other point x' of the polytope (see e.g. Definition 3.5 and Theorem 3.8 in [7]). In particular, $\omega \cdot \alpha < \omega \cdot \alpha'$ for all vertices $\alpha \neq \alpha'$.

For $y = t^\omega = \prod_{i=1}^n t^{\omega_i}$, we have

$$f(y) = \sum_{\alpha \in \mathbb{N}^n} c_\alpha (t^\omega)^\alpha = \sum_{\alpha \in \mathbb{N}^n} c_\alpha t^{\omega \cdot \alpha} = c_{\alpha'} t^{\omega \cdot \alpha'} + \sum_{\alpha \in \mathbb{N}^n, \alpha \neq \alpha'} c_\alpha t^{\omega \cdot \alpha}.$$

Now $f(y)$ is a well defined function for $t \in \mathbb{R}_{>0}$, which tends to $+\infty$ for t tending to infinity and $c_{\alpha'} > 0$ and to $-\infty$ for $c_{\alpha'} < 0$ (by assumption $c_{\alpha'} \neq 0$). Hence, by letting t be large enough, the sign of $f(y)$ agrees with the sign of $c_{\alpha'}$. □

Finding the vertices in practice. In the examples below, we find the vertices of the Newton polytope of the polynomial of interest as follows. We use Maple (version 2015). We construct first the polytope using the command *PolyhedralSet* and subsequently use the command *VerticesAndRays*, from the package *PolyhedralSets*, to find the vertices.

6 Details on the examples in the main text

6.1 Phosphorylation of two substrates

In this subsection we consider the network in the first row of Table 1 in the main text.

We consider a system in which two substrates can be either unphosphorylated, A, B or phosphorylated A_p, B_p. Phosphorylation of both substrates is catalyzed by the same kinase K and dephosphorylation of A_p, B_p is catalyzed by the same phosphatase F. That is, the system consists of two futile cycles sharing kinase and phosphatase.

The reactions of the system are:

$$A + K \underset{\kappa_2}{\overset{\kappa_1}{\rightleftharpoons}} AK \xrightarrow{\kappa_3} A_p + K \qquad B + K \underset{\kappa_8}{\overset{\kappa_7}{\rightleftharpoons}} BK \xrightarrow{\kappa_9} B_p + K$$

$$A_p + F \underset{\kappa_5}{\overset{\kappa_4}{\rightleftharpoons}} A_pF \xrightarrow{\kappa_6} A + F \qquad B_p + F \underset{\kappa_{11}}{\overset{\kappa_{10}}{\rightleftharpoons}} B_pF \xrightarrow{\kappa_{12}} B + F.$$

This network is a PTM network with substrates A, B, A_p, B_p, enzymes K, F and intermediates AK, BK, A_pF, B_pF. It was shown in [5] that this network with mass-action kinetics is multistationary. Here we find the necessary and sufficient condition on the reaction rate constants for having multistationarity in some stoichiometric compatibility class. We let

$$X_1 = K, \quad X_3 = A, \quad X_5 = B, \quad X_7 = AK, \quad X_9 = A_pF,$$
$$X_2 = F, \quad X_4 = A_p, \quad X_6 = B_p, \quad X_8 = BK, \quad X_{10} = B_pF.$$

The stoichiometric matrix N of the network and a row reduced matrix W whose rows from a basis of $\text{im}(N)^\perp$ are

$$N = \begin{pmatrix} -1 & 1 & 1 & 0 & 0 & 0 & -1 & 1 & 1 & 0 & 0 & 0 \\ 0 & 0 & 0 & -1 & 1 & 1 & 0 & 0 & 0 & -1 & 1 & 1 \\ -1 & 1 & 0 & 0 & 0 & 1 & 0 & 0 & 0 & 0 & 0 & 0 \\ 0 & 0 & 1 & -1 & 1 & 0 & 0 & 0 & 0 & 0 & 0 & 0 \\ 0 & 0 & 0 & 0 & 0 & 0 & -1 & 1 & 0 & 0 & 0 & 1 \\ 0 & 0 & 0 & 0 & 0 & 0 & 0 & 0 & 1 & -1 & 1 & 0 \\ 1 & -1 & -1 & 0 & 0 & 0 & 0 & 0 & 0 & 0 & 0 & 0 \\ 0 & 0 & 0 & 0 & 0 & 0 & 1 & -1 & -1 & 0 & 0 & 0 \\ 0 & 0 & 0 & 1 & -1 & -1 & 0 & 0 & 0 & 0 & 0 & 0 \\ 0 & 0 & 0 & 0 & 0 & 0 & 0 & 0 & 0 & 1 & -1 & -1 \end{pmatrix},$$

$$W = \begin{pmatrix} 1 & 0 & 0 & 0 & 0 & 0 & 1 & 1 & 0 & 0 \\ 0 & 1 & 0 & 0 & 0 & 0 & 0 & 0 & 1 & 1 \\ 0 & 0 & 1 & 1 & 0 & 0 & 1 & 0 & 1 & 0 \\ 0 & 0 & 0 & 0 & 1 & 1 & 0 & 1 & 0 & 1 \end{pmatrix}.$$

The rank of N is $s = 6$. The matrix W gives rise to the conservation relations

$$c_1 = x_1 + x_7 + x_8, \qquad c_3 = x_3 + x_4 + x_7 + x_9,$$
$$c_2 = x_2 + x_9 + x_{10}, \qquad c_4 = x_5 + x_6 + x_8 + x_{10},$$

where c_1, c_2, c_3, c_4 correspond to the total amounts of kinase, phosphatase, substrate A and substrate B, respectively.

With mass-action kinetics, the vector of reaction rates is

$$v(x) = (\kappa_1 x_1 x_3, \kappa_2 x_7, \kappa_3 x_7, \kappa_4 x_2 x_4, \kappa_5 x_9, \kappa_6 x_9, \kappa_7 x_1 x_5, \kappa_8 x_8, \kappa_9 x_8, \kappa_{10} x_2 x_6, \kappa_{11} x_{10}, \kappa_{12} x_{10}).$$

The function $f(x) = Nv(x)$ is thus

$$\begin{aligned} f(x) = (&-\kappa_1 x_1 x_3 - \kappa_7 x_1 x_5 + \kappa_2 x_7 + \kappa_3 x_7 + \kappa_8 x_8 + \kappa_9 x_8, \\ &- \kappa_4 x_2 x_4 - \kappa_{10} x_2 x_6 + \kappa_5 x_9 + \kappa_6 x_9 + \kappa_{11} x_{10} + \kappa_{12} x_{10}, -\kappa_1 x_1 x_3 + \kappa_2 x_7 + \kappa_6 x_9, \\ &- \kappa_7 x_1 x_5 + \kappa_8 x_8 + \kappa_{12} x_{10}, -\kappa_{10} x_2 x_6 + \kappa_9 x_8 + \kappa_{11} x_{10}, \kappa_1 x_1 x_3 - \kappa_2 x_7 - \kappa_3 x_7, \\ &\kappa_7 x_1 x_5 - \kappa_8 x_8 - \kappa_9 x_8, \kappa_4 x_2 x_4 - \kappa_5 x_9 - \kappa_6 x_9, \kappa_{10} x_2 x_6 - \kappa_{11} x_{10} - \kappa_{12} x_{10}). \end{aligned}$$

We apply the algorithm to this network with the matrix N and the vector $v(x)$.

Step 1. Mass-action kinetics fulfils assumption (12). The function $f(x)$ and W are given above and the matrix W is row reduced.

Step 2. The network is a PTM network, hence it is conservative and thus dissipative.

Step 3. We apply the reduction technique from Section 5.1. The network has four intermediates AK, A_pF, BK, B_pF. After their elimination, we are left with the reaction network

$$A + K \longrightarrow A_p + K \qquad B + K \longrightarrow B_p + K \qquad A_p + F \longrightarrow A + F \qquad B_p + F \longrightarrow B + F.$$

This network has two catalysts: K, F. Their elimination yields the reaction network (the so-called *underlying substrate network* in the main text)

$$A \rightleftharpoons A_p \qquad B \rightleftharpoons B_p.$$

This is a monomolecular network with two strongly connected components. By Corollary 5.3, there are no boundary equilibria in any $\mathcal{P}_c$ for which $\mathcal{P}_c^+ \neq \emptyset$.

Step 4. For our choice of W, we have $i_1 = 1, i_2 = 2, i_3 = 3, i_4 = 5$. The function $\varphi_c(x)$ is thus

$$\begin{aligned} \varphi_c(x) = \big(&x_1 + x_7 + x_8 - c_1, x_2 + x_9 + x_{10} - c_2, x_3 + x_4 + x_7 + x_9 - c_3, \\ &- \kappa_4 x_2 x_4 + \kappa_3 x_7 + \kappa_5 x_9, x_5 + x_6 + x_8 + x_{10} - c_4, -\kappa_{10} x_2 x_6 + \kappa_9 x_8 + \kappa_{11} x_{10}, \\ &\kappa_1 x_1 x_3 - \kappa_2 x_7 - \kappa_3 x_7, \kappa_7 x_1 x_5 - \kappa_8 x_8 - \kappa_9 x_8, \kappa_4 x_2 x_4 - \kappa_5 x_9 - \kappa_6 x_9, \\ &\kappa_{10} x_2 x_6 - \kappa_{11} x_{10} - \kappa_{12} x_{10}\big). \end{aligned}$$

The Jacobian matrix $M(x) = J_{\varphi_c}(x)$ is

$$\begin{pmatrix} 1 & 0 & 0 & 0 & 0 & 0 & 1 & 1 & 0 & 0 \\ 0 & 1 & 0 & 0 & 0 & 0 & 0 & 0 & 1 & 1 \\ 0 & 0 & 1 & 1 & 0 & 0 & 1 & 0 & 1 & 0 \\ 0 & -\kappa_4 x_4 & 0 & -\kappa_4 x_2 & 0 & 0 & \kappa_3 & 0 & \kappa_5 & 0 \\ 0 & 0 & 0 & 0 & 1 & 1 & 0 & 1 & 0 & 1 \\ 0 & -\kappa_{10} x_6 & 0 & 0 & 0 & -\kappa_{10} x_2 & 0 & \kappa_9 & 0 & \kappa_{11} \\ \kappa_1 x_3 & 0 & \kappa_1 x_1 & 0 & 0 & 0 & -\kappa_2 - \kappa_3 & 0 & 0 & 0 \\ \kappa_7 x_5 & 0 & 0 & 0 & \kappa_7 x_1 & 0 & 0 & -\kappa_8 - \kappa_9 & 0 & 0 \\ 0 & \kappa_4 x_4 & 0 & \kappa_4 x_2 & 0 & 0 & 0 & 0 & -\kappa_5 - \kappa_6 & 0 \\ 0 & \kappa_{10} x_6 & 0 & 0 & 0 & \kappa_{10} x_2 & 0 & 0 & 0 & -\kappa_{11} - \kappa_{12} \end{pmatrix}.$$

The determinant of $M(x)$ is a large polynomial. We omit it here.

Step 5. The determinant of $M(x)$ has terms of sign $(-1)^{s+1} = -1$. We postpone the discussion of the conditions on the reaction rate constants for which all terms have sign $(-1)^s$ to Step 7. We proceed to the next step.

Step 6. There is a non-interacting set with $s = 6$ species:

$$\{X_4, X_6, X_7, X_8, X_9, X_{10}\} = \{A_p, B_p, AK, BK, A_pF, B_pF\}.$$

By solving the equilibrium equations $f_4 = f_6 = f_7 = f_8 = f_9 = f_{10} = 0$ in the variables $x_4, x_6, x_7, x_8, x_9, x_{10}$, we find the following positive parameterization of the set of equilibria in terms of $\widehat{x} = (x_1, x_2, x_3, x_5)$:

$$x_4 = \frac{(\kappa_6 + \kappa_5)\,\kappa_3 x_3 x_1 \kappa_1}{\kappa_6\,(\kappa_3 + \kappa_2)\,x_2 \kappa_4}, \qquad x_7 = \frac{\kappa_1 x_1 x_3}{\kappa_3 + \kappa_2}, \qquad x_9 = \frac{\kappa_3 x_3 x_1 \kappa_1}{\kappa_6\,(\kappa_3 + \kappa_2)},$$

$$x_6 = \frac{(\kappa_{12} + \kappa_{11})\,\kappa_9 x_5 x_1 \kappa_7}{\kappa_{12}\,(\kappa_9 + \kappa_8)\,\kappa_{10} x_2}, \qquad x_8 = \frac{\kappa_7 x_1 x_5}{\kappa_9 + \kappa_8}, \qquad x_{10} = \frac{\kappa_9 x_5 x_1 \kappa_7}{\kappa_{12}\,(\kappa_9 + \kappa_8)}.$$

The free variables of the parameterization are the concentrations of the two enzymes and one substrate per conservation relation involving substrates.

Step 7. The function $a(\widehat{x})$ is a large rational function with positive denominator. Therefore, the numerator of this function, a polynomial $p(\widehat{x})$, determines the sign of $a(\widehat{x})$. The coefficients are polynomials in $\kappa_1, \dots, \kappa_{10}$. All but one of the coefficients are polynomials in $\kappa_1, \dots, \kappa_{10}$ with positive coefficients. Therefore, all coefficients but one are always positive, independently of the values of the reaction rate constants $\kappa_1, \dots, \kappa_{10}$.

The only coefficient with sign depending on the specific values of $\kappa_1, \dots, \kappa_{10}$ is

$$\alpha(\kappa) = \kappa_1\kappa_7\,(\kappa_3\kappa_{12} - \kappa_6\kappa_9)\,(\kappa_1\kappa_3\kappa_5\kappa_8\kappa_{10}\kappa_{12} + \kappa_1\kappa_3\kappa_5\kappa_9\kappa_{10}\kappa_{12} + \kappa_1\kappa_3\kappa_6\kappa_8\kappa_{10}\kappa_{12} + \kappa_1\kappa_3\kappa_6\kappa_9\kappa_{10}\kappa_{12} - \kappa_2\kappa_4\kappa_6\kappa_7\kappa_9\kappa_{11} - \kappa_2\kappa_4\kappa_6\kappa_7\kappa_9\kappa_{12} - \kappa_3\kappa_4\kappa_6\kappa_7\kappa_9\kappa_{11} - \kappa_3\kappa_4\kappa_6\kappa_7\kappa_9\kappa_{12}).$$

If $\alpha(\kappa) \geq 0$, then all coefficients of $p(\widehat{x})$ are positive, and hence $a(\widehat{x})$ is positive for all positive $\widehat{x}$. Using $(-1)^s = (-1)^6 = 1$, Corollary 4.2(A) (Corollary 2(A) in the main text) gives that there is a unique positive equilibrium in each stoichiometric compatibility class with non-empty positive relative interior.

When this coefficient is negative, then we need to check whether $p(\widehat{x})$ is negative for some $\widehat{x}$. We analyse this by finding the Newton polytope and using Proposition 5.4.

The coefficient $\alpha(\kappa)$ corresponds to the monomial $x_1^2 x_2 x_3 x_5$. The exponent vectors of the monomials of $p(\widehat{x})$ are:

$$(0,3,0,0),\ (0,3,0,1),\ (0,3,1,0),\ (1,2,0,0),\ (1,2,0,1),\ (1,2,1,0),\ (1,3,0,0),$$
$$(1,3,0,1)\ (1,3,1,0),\ (2,1,0,0),\ (2,1,0,1),\ (2,1,1,0),\ (2,1,1,1),\ (2,2,0,0),$$
$$(2,3,0,0),\ (3,0,0,1),\ (3,0,1,0),\ (3,1,0,1),\ (3,1,1,0).$$

We find the vertices of the convex hull of the exponent vectors, and find that they are

$$(0,3,0,1),\ (0,3,1,0),\ (\mathbf{2},\mathbf{1},\mathbf{1},\mathbf{1}),\ (0,3,0,0),\ (2,1,0,0),\ (3,0,0,1),$$
$$(3,0,1,0),\ (1,3,0,1),\ (1,3,1,0),\ (2,3,0,0),\ (3,1,0,1),\ (3,1,1,0).$$

Thus the exponent vector of the monomial of interest, $(2,1,1,1)$ (highlighted in bold), is a vertex of the Newton polytope. Therefore, by Proposition 5.4, there exists $\widehat{x}$ such that $p(\widehat{x})$ is negative. Corollary 4.2(B) (Corollary 2(B) in the main text) gives that there is a stoichiometric compatibility class with multiple positive equilibria.

The condition $\alpha(\kappa) < 0$ can be rewritten as:

$$(\kappa_3\kappa_{12} - \kappa_6\kappa_9)(\kappa_3\kappa_{12}\kappa_1\kappa_{10}(\kappa_5 + \kappa_6)(\kappa_8 + \kappa_9) - \kappa_6\kappa_9\kappa_4\kappa_7(\kappa_2 + \kappa_3)(\kappa_{11} + \kappa_{12})) < 0,$$

which in turn can be written as

$$(\kappa_3\kappa_{12} - \kappa_6\kappa_9)\left(\kappa_3\kappa_{12}\cdot\frac{\kappa_1}{\kappa_2+\kappa_3}\cdot\frac{\kappa_{10}}{\kappa_{11}+\kappa_{12}} - \kappa_6\kappa_9\cdot\frac{\kappa_4}{\kappa_5+\kappa_6}\cdot\frac{\kappa_7}{\kappa_8+\kappa_9}\right) < 0,$$

Note that $\kappa_3, \kappa_6, \kappa_9, \kappa_{12}$ are the catalytic constants of phosphorylation/dephosphorylation of A and B ($k_{c1}, k_{c2}, k_{c3}, k_{c4}$ in the main text), and

$$k_{M1}^{-1} = \frac{\kappa_1}{\kappa_2+\kappa_3}, \qquad k_{M2}^{-1} = \frac{\kappa_4}{\kappa_5+\kappa_6}, \qquad k_{M3}^{-1} = \frac{\kappa_7}{\kappa_8+\kappa_9}, \qquad k_{M4}^{-1} = \frac{\kappa_{10}}{\kappa_{11}+\kappa_{12}}$$

are the inverses of the Michaelis-Menten constants of K and F for each substrate. Therefore, the necessary and sufficient condition for multistationarity can be written in terms of the catalytic constants and the Michaelis-Menten constants,

$$(\kappa_3\kappa_{12} - \kappa_6\kappa_9)\left(\frac{\kappa_3\kappa_{12}}{k_{M1}k_{M4}} - \frac{\kappa_6\kappa_9}{k_{M2}k_{M3}}\right) < 0.$$

This proves the condition for multiple and unique equilibria given in the first row of Table 1 in the main text, by letting

$$k_{c1} = \kappa_3, \qquad k_{c2} = \kappa_6, \qquad k_{c3} = \kappa_9, \qquad k_{c4} = k_{12}.$$

In particular, we have that

- If $\kappa_3\kappa_{12} > \kappa_6\kappa_9$, then we need $\frac{\kappa_3\kappa_{12}}{k_{M1}k_{M4}} < \frac{\kappa_6\kappa_9}{k_{M2}k_{M3}}$ for multiple equilibria to occur.
- If $\kappa_3\kappa_{12} < \kappa_6\kappa_9$, then we need $\frac{\kappa_3\kappa_{12}}{k_{M1}k_{M4}} > \frac{\kappa_6\kappa_9}{k_{M2}k_{M3}}$ for multiple equilibria to occur.

6.2 Two-site phosphorylation system

In this subsection we consider the network in the second row of Table 1 in the main text. The conditions given here were also found in [3], the paper that lay the foundations of this algorithm. In this work we consider a direct route using the function φ_c and avoiding changes of variables. We explain here how to find the conditions using the algorithm in the main text.

We consider a system in which one substrate undergoes sequential and distributive phosphorylation by a kinase K and sequential and distributive dephosphorylation by a phosphatase F. The three phosphoforms of the substrate are A, A_p, A_{pp}. The reactions of the system are:

$$A + K \underset{\kappa_2}{\overset{\kappa_1}{\rightleftharpoons}} AK \xrightarrow{\kappa_3} A_p + K \underset{\kappa_8}{\overset{\kappa_7}{\rightleftharpoons}} A_pK \xrightarrow{\kappa_9} A_{pp} + K$$

$$A_{pp} + F \underset{\kappa_{11}}{\overset{\kappa_{10}}{\rightleftharpoons}} A_{pp}F \xrightarrow{\kappa_{12}} A_p + F \underset{\kappa_5}{\overset{\kappa_4}{\rightleftharpoons}} A_pF \xrightarrow{\kappa_6} A + F$$

This network is a PTM network with substrates A, A_p, A_{pp}, enzymes K, F and intermediates AK, A_pK, A_pF, A_{ppF}. We let

$$X_1 = K, \quad X_3 = A, \quad X_5 = A_{pp}, \quad X_6 = AK, \quad X_7 = A_pF,$$
$$X_2 = F, \quad X_4 = A_p, \quad X_8 = A_pK, \quad X_9 = A_{pp}F.$$

The stoichiometric matrix N of the network and a row reduced matrix W whose rows from a basis of $\mathrm{im}(N)^\perp$ are

$$N = \begin{pmatrix} -1 & 1 & 1 & 0 & 0 & 0 & -1 & 1 & 1 & 0 & 0 & 0 \\ 0 & 0 & 0 & -1 & 1 & 1 & 0 & 0 & 0 & -1 & 1 & 1 \\ -1 & 1 & 0 & 0 & 0 & 1 & 0 & 0 & 0 & 0 & 0 & 0 \\ 0 & 0 & 1 & -1 & 1 & 0 & -1 & 1 & 0 & 0 & 0 & 1 \\ 0 & 0 & 0 & 0 & 0 & 0 & 0 & 0 & 1 & -1 & 1 & 0 \\ 1 & -1 & -1 & 0 & 0 & 0 & 0 & 0 & 0 & 0 & 0 & 0 \\ 0 & 0 & 0 & 1 & -1 & -1 & 0 & 0 & 0 & 0 & 0 & 0 \\ 0 & 0 & 0 & 0 & 0 & 0 & 1 & -1 & -1 & 0 & 0 & 0 \\ 0 & 0 & 0 & 0 & 0 & 0 & 0 & 0 & 0 & 1 & -1 & -1 \end{pmatrix},$$

$$W = \begin{pmatrix} 1 & 0 & 0 & 0 & 0 & 1 & 0 & 1 & 0 \\ 0 & 1 & 0 & 0 & 0 & 0 & 1 & 0 & 1 \\ 0 & 0 & 1 & 1 & 1 & 1 & 1 & 1 & 1 \end{pmatrix}.$$

The rank of N is $s = 6$. The matrix W gives rise to the conservation relations

$$c_1 = x_1 + x_6 + x_8, \qquad c_2 = x_2 + x_7 + x_9, \qquad c_3 = x_3 + x_4 + x_5 + x_6 + x_7 + x_8 + x_9,$$

where c_1, c_2, c_3 correspond to the total amounts of kinase, phosphatase and substrate A, respectively.

With mass-action kinetics, the vector of reaction rates is

$$v(x) = (\kappa_1 x_1 x_3, \kappa_2 x_6, \kappa_3 x_6, \kappa_4 x_2 x_4, \kappa_5 x_7, \kappa_6 x_7, \kappa_7 x_1 x_4, \kappa_8 x_8, \kappa_9 x_8, \kappa_{10} x_2 x_5, \kappa_{11} x_9, \kappa_{12} x_9).$$

The function $f(x) = Nv(x)$ is thus

$$\begin{aligned} f(x) = (&-\kappa_1 x_1 x_3 - \kappa_7 x_1 x_4 + \kappa_2 x_6 + \kappa_3 x_6 + \kappa_8 x_8 + \kappa_9 x_8, \\ &- \kappa_4 x_2 x_4 - \kappa_{10} x_2 x_5 + \kappa_5 x_7 + \kappa_6 x_7 + \kappa_{11} x_9 + \kappa_{12} x_9, -\kappa_1 x_1 x_3 + \kappa_2 x_6 + \kappa_6 x_7, \\ &- \kappa_4 x_2 x_4 - \kappa_7 x_1 x_4 + \kappa_3 x_6 + \kappa_5 x_7 + \kappa_8 x_8 + \kappa_{12} x_9, -\kappa_{10} x_2 x_5 + \kappa_9 x_8 + \kappa_{11} x_9, \\ &\kappa_1 x_1 x_3 - \kappa_2 x_6 - \kappa_3 x_6, \kappa_4 x_2 x_4 - \kappa_5 x_7 - \kappa_6 x_7, \kappa_7 x_1 x_4 - \kappa_8 x_8 - \kappa_9 x_8, \\ &\kappa_{10} x_2 x_5 - \kappa_{11} x_9 - \kappa_{12} x_9). \end{aligned}$$

We apply the algorithm to this network with the matrix N and the vector $v(x)$.

Step 1. Mass-action kinetics fulfils assumption (12). The function $f(x)$ and W are given above and the matrix W is row reduced.

Step 2. This network is dissipative since it is a PTM network.

Step 3. The network has four intermediates $AK, A_pK, A_pF, A_{pp}F$. After their elimination, we are left with a reaction network with two catalysts: K, F. Their elimination yields the following underlying substrate network

$$A \rightleftharpoons A_p \rightleftharpoons A_{pp}.$$

This is a monomolecular network with two strongly connected components. By Corollary 5.3, there are no boundary equilibria in any $\mathcal{P}_c$ for which $\mathcal{P}_c^+ \neq \emptyset$.

Step 4. For our choice of W, we have $i_1 = 1, i_2 = 2, i_3 = 3$. The function $\varphi_c(x)$ is thus

$$\begin{aligned}\varphi_c(x) = \big(&x_1 + x_6 + x_8 - c_1, x_2 + x_7 + x_9 - c_2, x_3 + x_4 + x_5 + x_6 + x_7 + x_8 + x_9 - c_3,\\ &- \kappa_4 x_2 x_4 - \kappa_7 x_1 x_4 + \kappa_3 x_6 + \kappa_5 x_7 + \kappa_8 x_8 + \kappa_{12} x_9, -\kappa_{10} x_2 x_5 + \kappa_9 x_8 + \kappa_{11} x_9,\\ &\kappa_1 x_1 x_3 - \kappa_2 x_6 - \kappa_3 x_6, \kappa_4 x_2 x_4 - \kappa_5 x_7 - \kappa_6 x_7, \kappa_7 x_1 x_4 - \kappa_8 x_8 - \kappa_9 x_8,\\ &\kappa_{10} x_2 x_5 - \kappa_{11} x_9 - \kappa_{12} x_9\big).\end{aligned}$$

The Jacobian matrix $M(x) = J_{\varphi_c}(x)$ is

$$\begin{pmatrix} 1 & 0 & 0 & 0 & 0 & 1 & 0 & 1 & 0\\ 0 & 1 & 0 & 0 & 0 & 0 & 1 & 0 & 1\\ 0 & 0 & 1 & 1 & 1 & 1 & 1 & 1 & 1\\ -\kappa_7 x_4 & -\kappa_4 x_4 & 0 & -\kappa_4 x_2 - \kappa_7 x_1 & 0 & \kappa_3 & \kappa_5 & \kappa_8 & \kappa_{12}\\ 0 & -\kappa_{10} x_5 & 0 & 0 & -\kappa_{10} x_2 & 0 & 0 & \kappa_9 & \kappa_{11}\\ \kappa_1 x_3 & 0 & \kappa_1 x_1 & 0 & 0 & -\kappa_2 - \kappa_3 & 0 & 0 & 0\\ 0 & \kappa_4 x_4 & 0 & \kappa_4 x_2 & 0 & 0 & -\kappa_5 - \kappa_6 & 0 & 0\\ \kappa_7 x_4 & 0 & 0 & \kappa_7 x_1 & 0 & 0 & 0 & -\kappa_8 - \kappa_9 & 0\\ 0 & \kappa_{10} x_5 & 0 & 0 & \kappa_{10} x_2 & 0 & 0 & 0 & -\kappa_{11} - \kappa_{12} \end{pmatrix}.$$

The determinant of $M(x)$ is a large polynomial. We omit it here.

Step 5. The determinant of $M(x)$ has terms of sign $(-1)^{s+1} = -1$. We postpone the discussion of the conditions on the reaction rate constants for which all terms have sign $(-1)^s$ to Step 7. We proceed to the next step.

Step 6. This network is a PTM network and has a non-interacting set with $s = 6$ species:

$$\{X_4, X_5, X_6, X_7, X_8, X_9\} = \{A_p, A_{pp}, AK, A_pF, A_pK, A_{pp}F\}.$$

By solving the equilibrium equations $f_4 = f_5 = f_6 = f_7 = f_8 = f_9 = 0$ in the variables $x_4, \dots, x_9$, we find the following positive parameterization of the set of equilibria in terms of $\widehat{x} = (x_1, x_2, x_3)$:

$$x_4 = \frac{\kappa_1\kappa_3(\kappa_5+\kappa_6)x_1x_3}{(\kappa_2+\kappa_3)\kappa_4\kappa_6 x_2}, \qquad x_5 = \frac{\kappa_1\kappa_3(\kappa_5+\kappa_6)\kappa_7\kappa_9(\kappa_{11}+\kappa_{12})x_1^2x_3}{(\kappa_2+\kappa_3)\kappa_4\kappa_6(\kappa_8+\kappa_9)\kappa_{10}\kappa_{12}x_2^2},$$

$$x_6 = \frac{\kappa_1 x_1 x_3}{\kappa_2+\kappa_3}, \qquad x_7 = \frac{\kappa_1\kappa_3 x_1 x_3}{(\kappa_2+\kappa_3)\kappa_6},$$

$$x_8 = \frac{\kappa_1\kappa_3(\kappa_5+\kappa_6)\kappa_7 x_1^2 x_3}{\kappa_2+\kappa_3)\kappa_4\kappa_6(\kappa_8+\kappa_9)x_2}, \qquad x_9 = \frac{\kappa_1\kappa_3(\kappa_5+\kappa_6)\kappa_7\kappa_9 x_1^2 x_3}{\kappa_2+\kappa_3)\kappa_4\kappa_6(\kappa_8+\kappa_9)\kappa_{12}x_2}.$$

The free variables of this parameterization are the concentrations of the two enzymes and one of the substrates. We substitute $x_4, \dots, x_9$ with their expressions in the parameterization in $\det(M(x))$ to find $a(\widehat{x})$. The function $a(\widehat{x})$ is a large rational function with positive denominator which we do not include here.

Step 7. The numerator of $a(\widehat{x})$, the polynomial $p(\widehat{x})$, determines therefore the sign of $a(\widehat{x})$. The coefficients are polynomials in $\kappa_1, \dots, \kappa_{10}$.

The polynomial has 15 terms, 9 of which are positive for all values of the reaction rate constants. The remaining 6 coefficients are polynomials in $\kappa_1, \dots, \kappa_{10}$ that can either be positive or negative.

Five of the six coefficients are of the form $\beta(\kappa)b_1(\kappa)$, where $\beta(\kappa)$ is a positive polynomial in κ and

$$b_1(\kappa) = \kappa_3\kappa_{12} - \kappa_6\kappa_9$$

(thus $b_1(\kappa)$ is the same for all five coefficients). These five coefficients correspond to the monomials $x_1^3x_2^2x_3$, $x_1^2x_2^2x_3^2$, $x_1^3x_2x_3^2$, $x_1^2x_2^3x_3$ and $x_1^4x_3^2$.

The remaining coefficient is of the form $\gamma(\kappa)\alpha(\kappa)$, where $\gamma(\kappa)$ is a positive polynomial in κ and

$$\alpha(\kappa) = \kappa_1\kappa_3\kappa_4\kappa_8\kappa_{10}\kappa_{12} + \kappa_1\kappa_3\kappa_4\kappa_9\kappa_{10}\kappa_{12} + \kappa_1\kappa_3\kappa_5\kappa_7\kappa_{10}\kappa_{12} + \kappa_1\kappa_3\kappa_6\kappa_7\kappa_{10}\kappa_{12} - \kappa_1\kappa_4\kappa_6\kappa_7\kappa_9\kappa_{11} - \kappa_1\kappa_4\kappa_6\kappa_7\kappa_9\kappa_{12} - \kappa_2\kappa_4\kappa_6\kappa_7\kappa_9\kappa_{10} - \kappa_3\kappa_4\kappa_6\kappa_7\kappa_9\kappa_{10}.$$

It corresponds to the monomial $x_1^2x_2^2x_3$.

Since $(-1)^6 = 1$, part Corollary 4.2(A) (Corollary 2(A) in the main text) tells us that there is a unique positive equilibrium in each stoichiometric compatibility class with non-empty positive part, if

$$b_1(\kappa) \geq 0 \qquad \text{and} \qquad \alpha(\kappa) \geq 0.$$

The condition $\alpha(\kappa) \geq 0$ can be rewritten as:

$$\kappa_1\kappa_3\kappa_{10}\kappa_{12}\big(\kappa_4(\kappa_9+\kappa_8)+\kappa_7(\kappa_6+\kappa_5)\big) - \kappa_4\kappa_6\kappa_7\kappa_9\big(\kappa_1(\kappa_{12}+\kappa_{11})+\kappa_{10}(\kappa_3+\kappa_2)\big) \geq 0.$$

Dividing the expression by $\kappa_1\kappa_4\kappa_7\kappa_{10}$, the condition can be rewritten as

$$\kappa_3\kappa_{12}\big(k_{M2}+k_{M3}\big) - \kappa_6\kappa_9\big(k_{M1}+k_{M4}\big) \geq 0,$$

where

$$k_{M1} = \frac{\kappa_2+\kappa_3}{\kappa_1}, \qquad k_{M2} = \frac{\kappa_5+\kappa_6}{\kappa_4}, \qquad k_{M3} = \frac{\kappa_8+\kappa_9}{\kappa_7}, \qquad k_{M4} = \frac{\kappa_{11}+\kappa_{12}}{\kappa_{10}}$$

are the Michaelis-Menten constants of K and F for each site. Note that $\kappa_3, \kappa_6, \kappa_9, \kappa_{12}$ are the catalytic constants of phosphorylation of A, dephosphorylation of A_p, phosphorylation of A_p and dephosphorylation of A_{pp}. These are denoted by $k_{c1}, k_{c2}, k_{c3}, k_{c4}$ in the main text by letting

$$k_{c1} = \kappa_3, \qquad k_{c2} = \kappa_6, \qquad k_{c3} = \kappa_9, \qquad k_{c4} = k_{12}.$$

By letting

$$b_2(\kappa) = \kappa_3\kappa_{12}\big(k_{M2}+k_{M3}\big) - \kappa_6\kappa_9\big(k_{M1}+k_{M4}\big),$$

$\alpha(\kappa) \geq 0$ if and only if $b_2(\kappa) \geq 0$. Thus we have proven the condition for unique equilibria given in the second row of Table 1 in the main text.

Let us consider whether Corollary 4.2(B) (Corollary 2(B) in the main text) applies if $b_1(\kappa) < 0$ and/or $\alpha(\kappa) < 0$. The exponent vectors of the monomials of $p(\widehat{x})$ are:

$$\begin{matrix} (3,1,1) & (1,3,1) & (2,2,1) & (2,2,2) & (2,3,0) & (2,2,0) & (1,3,0) & (3,1,2) \\ (2,3,1) & (3,2,1) & (4,0,2) & (4,0,1) & (0,4,1) & (1,4,0) & (0,4,0) & \end{matrix}$$

The vertices of the convex hull of the exponent vectors are

$$(2,3,0) \quad (4,0,1) \quad (2,2,0) \quad (0,4,0) \quad (1,4,0) \quad (\mathbf{3},\mathbf{2},\mathbf{1}) \quad (4,0,2) \quad (0,4,1) \quad (2,3,1) \quad (2,2,2).$$

The vertex highlighted in bold corresponds to the monomial $x_1^3x_2^2x_3$, whose sign depends on $b_1(\kappa)$. By Proposition 5.4, if $b_1(\kappa) < 0$, then there exists $\widehat{x}$ such that $p(\widehat{x})$ is negative. Corollary 4.2(B) (Corollary 2(B) in the main text) gives that there is a stoichiometric compatibility class that admits positive multiple equilibria. This proves the condition for multistationarity given in the second row of Table 1 in the main text.

The exponent vector of the monomial corresponding to the coefficient $\alpha(\kappa)$, $(2,2,1)$, is not a vertex of the Newton polytope. In this case it is uncertain whether the condition $\alpha(\kappa) < 0$ is sufficient for multistationarity.

6.3 Two-substrate enzyme catalysis

This section contains an additional example to illustrate the application of the algorithm to a monostationary network for which a parameterization is required to reach the conclusion.

We consider a mechanism in which an enzyme E binds two substrates, S_1, S_2, in an unordered manner in order to catalyze the reversible conversion to the product P. A variation of this system was considered in [4]. The reactions of the system are:

$$E + S_1 \underset{\kappa_2}{\overset{\kappa_1}{\rightleftharpoons}} ES_1 \qquad S_2 + ES_1 \underset{\kappa_6}{\overset{\kappa_5}{\rightleftharpoons}} ES_1S_2 \qquad ES_1S_2 \underset{\kappa_8}{\overset{\kappa_7}{\rightleftharpoons}} E + P$$
$$E + S_2 \underset{\kappa_4}{\overset{\kappa_3}{\rightleftharpoons}} ES_2 \qquad S_1 + ES_2 \underset{\kappa_{10}}{\overset{\kappa_9}{\rightleftharpoons}} ES_1S_2.$$

We let

$$X_1 = E, \quad X_2 = S_1, \quad X_3 = ES_1, \quad X_4 = S_2, \quad X_5 = ES_2, \quad X_6 = ES_1S_2, \quad X_7 = P.$$

The stoichiometric matrix N of the network and a row reduced matrix W whose rows from a basis of $\mathrm{im}(N)^\perp$ are

$$N = \begin{pmatrix} -1 & 1 & -1 & 1 & 0 & 0 & 0 & 0 & 1 & -1 \\ -1 & 1 & 0 & 0 & 0 & 0 & 1 & -1 & 0 & 0 \\ 1 & -1 & 0 & 0 & -1 & 1 & 0 & 0 & 0 & 0 \\ 0 & 0 & -1 & 1 & -1 & 1 & 0 & 0 & 0 & 0 \\ 0 & 0 & 1 & -1 & 0 & 0 & 1 & -1 & 0 & 0 \\ 0 & 0 & 0 & 0 & 1 & -1 & -1 & 1 & -1 & 1 \\ 0 & 0 & 0 & 0 & 0 & 0 & 0 & 0 & 1 & -1 \end{pmatrix}$$

$$W = \begin{pmatrix} 1 & 0 & 1 & 0 & 1 & 1 & 0 \\ 0 & 1 & 1 & 0 & 0 & 1 & 1 \\ 0 & 0 & 0 & 1 & 1 & 1 & 1 \end{pmatrix}.$$

The rank of N is $s = 4$. The matrix W gives rise to the conservation relations

$$c_1 = x_1 + x_3 + x_5 + x_6, \qquad c_2 = x_2 + x_3 + x_6 + x_7 \qquad c_3 = x_4 + x_5 + x_6 + x_7,$$

where c_1, c_2, c_3, c_4 correspond to the total amounts of kinase, substrate S_1 and substrate S_2, respectively.

With mass-action kinetics, the vector of reaction rates is

$$v(x) = (\kappa_1 x_1 x_2, \kappa_2 x_3, \kappa_3 x_1 x_4, \kappa_4 x_5, \kappa_5 x_4 x_3, \kappa_6 x_6, \kappa_7 x_6, \kappa_8 x_2 x_5, \kappa_9 x_6, \kappa_{10} x_1 x_7).$$

The function $f(x) = Nv(x)$ is

$$\begin{aligned} f(x) = (&-\kappa_1 x_1 x_2 - \kappa_3 x_1 x_4 - \kappa_{10} x_1 x_7 + \kappa_2 x_3 + \kappa_4 x_5 + \kappa_9 x_6, \\ &- \kappa_1 x_1 x_2 - \kappa_8 x_2 x_5 + \kappa_2 x_3 + \kappa_7 x_6, \kappa_1 x_1 x_2 - \kappa_5 x_4 x_3 - \kappa_2 x_3 + \kappa_6 x_6 \\ &- \kappa_3 x_1 x_4 - \kappa_5 x_4 x_3 + \kappa_4 x_5 + \kappa_6 x_6, \kappa_3 x_1 x_4 - \kappa_8 x_2 x_5 - \kappa_4 x_5 + \kappa_7 x_6, \\ &\kappa_5 x_4 x_3 + \kappa_8 x_2 x_5 + \kappa_{10} x_1 x_7 - \kappa_6 x_6 - \kappa_7 x_6 - \kappa_9 x_6, -\kappa_{10} x_1 x_7 + \kappa_9 x_6). \end{aligned}$$

We apply the algorithm to this network with the matrix N and the vector $v(x)$.

Step 1. Mass-action kinetics fulfils assumption (12). The function $f(x)$ and W are given above and the matrix W is row reduced.

Step 2. The network is conservative since the concentration of every species is in the support of a conservation relation with positive coefficients. Therefore the network is dissipative.

Step 3. This network has only one intermediate ES_1S_2. Its removal yields the reaction network

$$\begin{array}{lll} E+S_1 \rightleftharpoons ES_1 & S_2+ES_1 \rightleftharpoons E+P & S_2+ES_1 \rightleftharpoons S_1+ES_2 \\ E+S_2 \rightleftharpoons ES_2 & S_1+ES_2 \rightleftharpoons E+P. & \end{array}$$

The conservation relations of this new network are (with the notation above):

$$c_1 = x_1 + x_3 + x_5, \qquad c_2 = x_2 + x_3 + x_7 \qquad c_3 = x_4 + x_5 + x_7.$$

The minimal siphons of the network are

$$\{E, ES_1, ES_2\}, \{S_1, ES_1, P\}, \{S_2, ES_2, P\}.$$

These siphons contain the support of the conservation relations for c_1, c_2, c_3 respectively. Thus, by Proposition 2 in the main text and Proposition 5.1, the original network does not have boundary equilibria in any stoichiometric compatibility class that intersects the positive orthant.

Step 4. For our choice of W, we have $i_1 = 1, i_2 = 2, i_3 = 4$. The function $\varphi_c(x)$ is thus

$$\begin{aligned} \varphi_c(x) = \Big(& x_1 + x_3 + x_5 + x_6 - c_1, x_2 + x_3 + x_6 + x_7 - c_2, \kappa_1 x_1 x_2 - \kappa_5 x_4 x_3 - \kappa_2 x_3 + \kappa_6 x_6, \\ & x_4 + x_5 + x_6 + x_7 - c_3, \kappa_3 x_1 x_4 - \kappa_8 x_2 x_5 - \kappa_4 x_5 + \kappa_7 x_6, \\ & \kappa_5 x_4 x_3 + \kappa_8 x_2 x_5 + \kappa_{10} x_1 x_7 - \kappa_6 x_6 - \kappa_7 x_6 - \kappa_9 x_6, -\kappa_{10} x_1 x_7 + \kappa_9 x_6 \Big). \end{aligned}$$

The Jacobian matrix $M(x) = J_{\varphi_c}(x)$ is

$$\begin{pmatrix} 1 & 0 & 1 & 0 & 1 & 1 & 0 \\ 0 & 1 & 1 & 0 & 0 & 1 & 1 \\ \kappa_1 x_2 & \kappa_1 x_1 & -\kappa_5 x_4 - \kappa_2 & -\kappa_5 x_3 & 0 & \kappa_6 & 0 \\ 0 & 0 & 0 & 1 & 1 & 1 & 1 \\ \kappa_3 x_4 & -\kappa_8 x_5 & 0 & \kappa_3 x_1 & -\kappa_8 x_2 - \kappa_4 & \kappa_7 & 0 \\ \kappa_{10} x_7 & \kappa_8 x_5 & \kappa_5 x_4 & \kappa_5 x_3 & \kappa_8 x_2 & -\kappa_6 - \kappa_7 - \kappa_9 & \kappa_{10} x_1 \\ -\kappa_{10} x_7 & 0 & 0 & 0 & 0 & \kappa_9 & -\kappa_{10} x_1 \end{pmatrix}.$$

The determinant of $M(x)$ is a large polynomial. We omit it here.

Step 5. The determinant of $M(x)$ has terms of sign $(-1)^{s+1} = -1$. We postpone the discussion of the conditions on the reaction rate constants for which all terms have sign $(-1)^s$ to Step 7. We proceed to the next step.

Step 6. This network is not a PTM system, but has a non-interacting set with $s = 4$ species:

$$\{X_3, X_5, X_6, X_7\} = \{ES_1, ES_2, ES_1S_2, P\}.$$

By solving the equilibrium equations $f_3 = f_5 = f_6 = f_7 = 0$ in the variables x_3, x_5, x_6, x_7, we

find the following positive parameterization of the set of equilibria in terms of $\widehat{x} = (x_1, x_2, x_4)$:

$$x_3 = \frac{x_2 x_1 \left(\kappa_1\kappa_6\kappa_8 x_2 + \kappa_3\kappa_6\kappa_8 x_4 + \kappa_1\kappa_4\kappa_6 + \kappa_1\kappa_4\kappa_7\right)}{\kappa_2\kappa_6\kappa_8 x_2 + \kappa_4\kappa_5\kappa_7 x_4 + \kappa_2\kappa_4\kappa_6 + \kappa_2\kappa_4\kappa_7},$$
$$x_5 = \frac{x_1 x_4 \left(\kappa_1\kappa_5\kappa_7 x_2 + \kappa_3\kappa_5\kappa_7 x_4 + \kappa_2\kappa_3\kappa_6 + \kappa_2\kappa_3\kappa_7\right)}{\kappa_2\kappa_6\kappa_8 x_2 + \kappa_4\kappa_5\kappa_7 x_4 + \kappa_2\kappa_4\kappa_6 + \kappa_2\kappa_4\kappa_7},$$
$$x_6 = \frac{x_2 x_4 \left(\kappa_1\kappa_5\kappa_8 x_2 + \kappa_3\kappa_5\kappa_8 x_4 + \kappa_1\kappa_4\kappa_5 + \kappa_2\kappa_3\kappa_8\right) x_1}{\kappa_2\kappa_6\kappa_8 x_2 + \kappa_4\kappa_5\kappa_7 x_4 + \kappa_2\kappa_4\kappa_6 + \kappa_2\kappa_4\kappa_7},$$
$$x_7 = \frac{\kappa_9 x_2 x_4 \left(\kappa_1\kappa_5\kappa_8 x_2 + \kappa_3\kappa_5\kappa_8 x_4 + \kappa_1\kappa_4\kappa_5 + \kappa_2\kappa_3\kappa_8\right)}{\left(\kappa_2\kappa_6\kappa_8 x_2 + \kappa_4\kappa_5\kappa_7 x_4 + \kappa_2\kappa_4\kappa_6 + \kappa_2\kappa_4\kappa_7\right)\kappa_{10}}.$$

We substitute x_3, x_5, x_6, x_7 with their expressions in the parameterization in $\det(M(x))$ to find $a(\widehat{x})$. The function $a(\widehat{x})$ is a large rational function.

Step 7. The numerator and denominator of $a(\widehat{x})$ are polynomials in x and κ with all coefficients positive. By Corollary 4.2(A) (Corollary 2(A) in the main text) using $s = 4$, there is a unique positive equilibrium in each stoichiometric compatibility class that intersects the positive orthant.

References

[1] H. Amann, *Ordinary differential equations: An introduction to nonlinear analysis*, De Gruyter studies in mathematics, de Gruyter, 1990.

[2] A Ben-Israel, *Notes on linear inequalities, I: The intersection of the nonnegative orthant with complementary orthogonal subspaces*, J. Math. Anal. Appl. **9** (1964), 303314.

[3] Carsten Conradi and Maya Mincheva, *Catalytic constants enable the emergence of bistability in dual phosphorylation*, J. R. S. Interface **11** (2014), no. 95.

[4] Gheorghe Craciun, Yangzhong Tang, and Martin Feinberg, *Understanding bistability in complex enzyme-driven reaction networks*, Proceedings of the National Academy of Sciences **103** (2006), no. 23, 8697–8702.

[5] Elisenda Feliu and Carsten Wiuf, *Enzyme-sharing as a cause of multi-stationarity in signalling systems*, Journal of The Royal Society Interface (2011), rsif20110664.

[6] Josef Hofbauer, *An index theorem for dissipative semiflows*, Rocky Mountain J. Math. **20** (1990), 1017–1031.

[7] M. Joswig and T. Theobald, *Polyhedral and algebraic methods in computational geometry*, Universitext, Springer, London, 2013.

[8] Patrick Leenheer, David Angeli, and Eduardo Sontag, *Monotone chemical reaction networks*, Journal of Mathematical Chemistry **41** (2007), 295–314.

[9] M. Marcondes de Freitas, E. Feliu, and C. Wiuf, *Intermediates, catalysts, persistence, and boundary steady states*, J. Math. Biol. **74** (2017), 887–932.

[10] R. T. Rockafellar, *Convex analysis*, Princeton University Press, 1997.

[11] A. Shiu and B. Sturmfels, *Siphons in chemical reaction networks*, Bull. Math. Biol. **72** (2010), no. 6, 1448–1463.

[12] G. Teschl, *Topics in real and functional analysis*, `http://www.mat.univie.ac.at/~gerald/ftp/book-fa/`, 2004.

[13] R. Vandervorst, *Topological methods for nonlinear differential equations*, `www.few.vu.nl/~vdvorst/Lectures2014a.pdf`, 2014.
